# Ecofriendly hydrothermal synthesis of lemon peel derived multifunctional carbon quantum dots for biomedical and environmental sensing applications

**DOI:** 10.1186/s11671-026-04524-7

**Published:** 2026-04-13

**Authors:** Gopika Venugopal, Riya Alex, K. Sreekanth, R. Anjali, B. Unnikrishnan, Divya Mathew, M. Smruthy, E. K. Radhakrishnan, Poulomi Mukherjee

**Affiliations:** 1 School of Biosciences, Mahatma Gandhi University, Kottayam, Kerala 686560 India; 2 Dr N Radhakrishnan International Centre for Medical Innovation, Mahatma Gandhi University, Kottayam, Kerala 686560 India; 3 Nuclear Agriculture and Biotechnology Division, Bhabha Atomic Research Centre, Mumbai, 400085 India; 4 Department of Atomic Energy, Homi Bhabha National Institute, Anushaktinagar, Mumbai, 400094 India

**Keywords:** Carbon quantum dots, Antimicrobial, Antibiofilm, Anti-inflammatory, Antidiabetic, Heavy metal sensing

## Abstract

In this study, carbon quantum dots were synthesized from lemon peel waste (LPCQDs) and were characterized by using UV-vis spectroscopy, FT-IR, HR-TEM, FE-SEM, DLS, ELS, fluorescence lifetime, quantum yield analysis, Raman spectroscopy, in-vitro cytotoxicity, and CHNS analysis. The average size of the LPCQDs synthesised in this study was 2.75 nm, with a quantum yield of 7.4% and a lifetime of 4.48 ns. In addition, the LPCQDs exhibited significant antimicrobial properties against a broad spectrum of clinical pathogens with a zone of inhibition up to 28 mm, an MIC value of 0.94 mg mL^− 1^ and biofilm inhibition of up to 81.72 ± 1.4%. The LPCQDs also resulted in the 96.07 ± 2.8% inhibition of protein denaturation, indicating a remarkable potential for anti-inflammatory applications. Further, LPCQDs demonstrated significant antidiabetic activity by inhibiting the activity of α-amylase by 65.8 ± 5.85%. The cytotoxicity assay results demonstrated promising biocompatibility of LPCQDs at concentrations up to 10 µg mL^− 1^. The LPCQDs also exhibited blue fluorescence under UV irradiation and exhibited fluorescence quenching in the presence of mercury and lead ions at concentrations as low as 700 µM, highlighting their suitability in metal ion detection systems.

**Graphical abstract:**

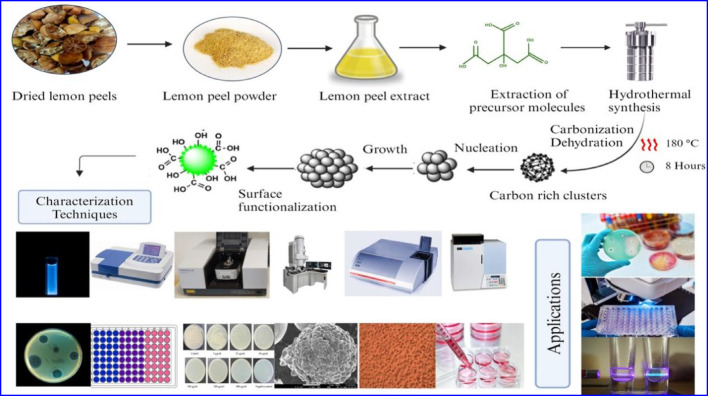

**Supplementary Information:**

The online version contains supplementary material available at 10.1186/s11671-026-04524-7.

## Highlights

- Carbon Quantum Dots of 2-4 nm were synthesized from lemon peel waste using an eco-friendly method.
- The synthesized LPCQDs exhibited excellent optical and physicochemical properties.
- LPCQDs demonstrated excellent antimicrobial, antibiofilm, anti-inflammatory, antidiabetic, and heavy metal sensing capabilities.
- Cytotoxicity analysis confirmed the biocompatibility of the synthesized LPCQDs.

**Supplementary Information:**

The online version contains supplementary material available at 10.1186/s11671-026-04524-7.

## Introduction

Rising population, urbanization, climate change, and waste mismanagement have led to the rapid deterioration of environmental health with risks to humans [1]. As per the current disease prediction patterns, existing treatment protocols are going to get outpaced by emerging pathogens. With the emerging understanding of “One Health”, environmental health has been considered to be intrinsically linked to human health, and a comprehensive approach is required to address the same [2]. Hence, a paradigm shift in the treatment strategies using sustainable materials is in high demand to address multiple health challenges, while offering the advantages of being hypoallergenic, naturally sourced, environmentally friendly, and with maximum efficacy at minimal dosage requirements [3]. With the advances in nanotechnology, many novel nanomaterials are being investigated for their therapeutic and remedial properties [4]. Several carbon based nanomaterials like carbon quantum dots (CQDs) have been synthesized and demonstrated to have applications in multidisciplinary areas due to their strong chemical bonding, versatile nature, and non-toxic properties [5]. The CQDs also stand out as a potential material due to their luminescent, quasi-spherical, and zero-dimensional properties [6]. The ultrafine size dimensions allow the easy surface modifiability of CQDs with higher stability and larger reactivity, along with the lower environmental impact [7]. However, the physicochemical synthesis of CQDs is often a very cumbersome or energy-demanding process, which can lead to a higher carbon footprint. Hence, the green synthesis of carbon dots using renewable and non-toxic precursors is considered to be a sustainable alternative to the existing conventional methods.

Plant derived materials have been used as potential precursors for the synthesis of quantum dots and are highly advantageous due to the presence of structurally diverse phytochemicals [8].The discarded peels of *Citrus limon*, abundant in bioactive metabolites, are an inexpensive and readily available waste material and hence have been selected as the precursor for the eco-friendly synthesis of CQDs in this investigation. Use of lemon peel waste as a carbon source forms a cost-effective approach to quantum dot synthesis and also results in the valorization of agricultural waste [9]. The lemon peel waste has several features favouring it to be a potential candidate for the carbon dot synthesis, which include the presence of higher percentages of carbonaceous compounds like citric acid, antioxidants [10], reductants, and capping agents [11]. Moreover, lemon peel extracts have been known to possess bioactive compounds like limonoids, flavonoids, and terpenes, which are rich in surface active functional groups that are important for the carbon dot formation and stability [12]. Lemon peel extracts have also been revealed in a previous study to have capping and reducing agents that could help to control the size of titanium nanoparticles [13]. Previous reports on carbon dots derived from lemon peel waste have also demonstrated them to have good blue emissive properties with comparable size and photostability, which allows them to be stored for longer time periods [14]. Previously reported studies of lemon peel derived CQDs have demonstrated the role of pretreatment steps in shaping the structure and composition of the carbon dots, which in turn affect their biocompatibility, optical and physicochemical properties [15,16]. Hence, the present study proposes a novel greener synthesis route by using safer chemicals for LPCQDs synthesis. Most of the previously reported lemon peel-derived CQDs rely on high-temperature (> 300 °C) hydrothermal treatments, harsh and corrosive chemical pretreatments, or involve multiple solvents. These approaches compromise sustainability, scalability, and biocompatibility. The method reported in this manuscript employs a sonication-assisted ethanolic extraction, followed by a hydrothermal treatment at a comparatively lower temperature (180 °C). Additionally, the use of minimally processed lemon peel extracts rich in intrinsic bioactive metabolites serves as the carbon source, reducing agent, and surface functionalizing matrix. Whereas in previous studies, surface functionalization is introduced post-synthesis through doping, the present approach uses in situ functionalization without additional modification steps. This makes the sustainable synthesis of multi-functional CQDs from lemon peel waste as conducted in this study, to be significant for both the valorization of wastes and green nanotechnology [17].

Green-synthesized CQDs have also been reported to have applications in the development of nanosensors, photocatalytic degradation, metal ion sensing, shelf-life enhancement and active packaging applications [18]. In the case of pathogenesis of inflammatory mechanisms, the potential of several carbon dots are to be explored in detail [19]. Moreover, a significant research gap exists in understanding the interactions of green-synthesized CQDs with biofilms formed by pathogens and also their action against different types of microorganisms. Also, in the present study, the LPCQDs were evaluated for their antimicrobial and antibiofilm potential against common clinical pathogens by using FE-SEM and AFM-based methods, which provides a new understanding on the broad spectrum of action of these carbon dots.

The multidimensional properties of CQDs make it a promising candidate for environmental applications. The release of toxic heavy metals into the water bodies can adversely affect human health, as these are non-biodegradable and are likely to have correlation with conditions like cancer due to bioaccumulation [20,21,22]. However, their detection at the ultralow level is highly challenging, which also offers opportunities for the development of CQD-based sensors [23]. The quenching effect of the CQDs has also been previously explored for applications related to the fabrication of metal based sensors, thermometry, photocatalytic degradation, and environmental monitoring [24]. Hence, the detailed characterisation of the lemon peel extract integrated into the comprehensive evaluation of the biological and environmental/sensing properties of the LPCQDs uniquely highlights the efficacy of the synthesis route and the versatility of the carbon dots formed.

## Methodology

### Materials

The solvents hexane and ethyl acetate used in the study were purchased from Merck Life Science Pvt Ltd., Mueller Hinton broth (MHB), potato dextrose broth (PDB), nutrient agar (NA), bovine serum albumin (BSA), egg albumin, and quercetin were purchased from Hi-Media Laboratories Pvt. Ltd. The standard strains of *Staphylococcus aureus*,* Escherichia coli*,* Salmonella typhi*,* Proteus mirabilis*,* Klebsiella pneumoniae*,* Candida albicans*,* Candida tropicalis* and *Aspergillus flavus* were acquired from the culture collection of the molecular microbiology laboratory at the School of Biosciences, Mahatma Gandhi University, Kerala. The L929 mouse fibroblast cell lines were procured from the National Centre for Cell Sciences (NCCS), Pune, India.

### Preparation of lemon peel extract (LPE) and screening for antibacterial activity

Here, peels of the fruit of *Citrus limon* (L.) *Osbeck* were procured from the local market at Ettumanoor, Kerala, India (Latitude: 9° 40’ 12.00” N, Longitude: 76° 34’ 12.00” E). Then, 6 g of shade dried peels were powdered and suspended in 90 mL of ethanol, and the solution was probe sonicated for 1.5 h at 95 ppm. The liquid suspension was centrifuged for 10 min at 8000×g, and the supernatant so obtained was passed through a 0.22 μm filter [25]. The inhibitory effect of LPCQDs on selected pathogens was examined using the standard well diffusion technique according to the CLSI guidelines. For this, *S. aureus* and *E. coli* strains were grown in nutrient broth overnight at 37 ℃. The OD of culture was then adjusted to 0.1OD at 600 nm using an Eppendorf biospectrophotometer D30. Wells of 8 mm diameter were further punctured on the MHA media under aseptic conditions using a gel borer, and the agar media were swabbed with the bacterial broth by the lawn culture method [26]. Then 60 µL of the lemon peel extract was micropipetted into the corresponding wells, and after overnight incubation, the zone of inhibition was measured [27,28].

### Characterization of prepared LPE

Here, the Gas Chromatography- Mass Spectrometry (GC-MS) analysis of LPE was carried out to profile the compounds present in it. The GC-MS system contained a flame ionization detector equipped with a stable phase, minimal bleed, DB-5 column (length: 30 m, inner diameter: 0.25 mm, and film thickness: 0.25 μm). Helium gas at 64 kPa pressure and constant flow rate of 1 mL min^−1^ was used as carrier phase, temperature was set at 60 °C for 2 min, succeeded by a 6 °C climb per min to 27 °C and a 10 min hold with the detector and injector temperature kept at 270 °C. The mass spectrum was scanned at 70 eV electron energy in the range of 50-500 amu as described before [29].

### Green synthesis of carbon quantum dots

For this, 20 mL of LPE was transferred into a mini hydrothermal autoclave and heated for 8 h at 180 °C. The liquid was then cooled to room temperature (RT) and further centrifuged at 8000 ×g for 10 min and syringe filtered with a 0.22 μm membrane, after which the filtrate was left at 60 °C for 5 h [24] to evaporate the ethanol content. Ethyl acetate and hexane were simultaneously added drop by drop for the precipitation of CQDs, and the precipitate was then dried at 60 °C to obtain the LPCQDs, which were suspended in 20 mL of distilled water [25].

### Characterization of synthesized LPCQDs

The spectroscopic study of LPCQDs was carried out by using the Shimadzu UV 2600 series spectrophotometer at a wavelength range of 200 to 800 nm [30]. The absolute quantum yield (QY) and photoluminescence lifetime of synthesized LPCQDs were measured by using the Horiba Fluorolog fluorescence spectrometer with an excitation wavelength of the excitation at 350 nm and an emission at 450 nm. The photoluminescence (PL) studies were conducted by recording the spectra of LPCQDs with scanning excitation wavelength from 200 to 430 nm (1 nm step) at a fixed emission wavelength of 450 nm in a black bottom 96-well plate after 12 months of storage under dark conditions. Parameters included 5 nm excitation bandwidth, 100 ms integration time, and automatic dynamic range using SkanIt Software v7.0. The Raman spectroscopic analysis of the synthesized LPCQDs was further performed by using the WITEC alpha300 RA Raman AFM microscope to determine the extent of structural defects present in the synthesized LPCQDs. The FTIR spectroscopic analysis of both the LPCQDs and LPE was also analysed by using the Perkin Elmer FT-IR spectrometer in attenuated total reflectance mode by using a contact sampling technique at a range 40,000-450 cm^-1^ and scan rate 4 cm^-1^ [30].

The TEM images of LPCQDs were obtained from the JEOL JEM 2100 h (LaB6) TYPE microscope, which was operated at 200 kV. For this, the samples were sonicated for 5 min, and a drop of the diluted sample was placed onto a carbon-coated TEM grid and used for the analysis [31]. The particle size distribution of the prepared LPCQDs was analyzed by using a Nanoparticle analyzer (HORIBA SZ-100) having the dynamic light scattering (DLS) functioning at 25 °C in an insulated chamber. Here, the light source was provided by the DPSS LASER at 532 nm. Additionally, the surface charge of particles (zeta-potential) was also measured under an electric field by using the same instrument. The elemental analysis of the LPCQDs was further performed by using an Elementar CHNS analyzer for the percentage composition analysis of elements such as C, H, N, and S in the sample [32].

### Antibacterial activity of LPCQDs

The antibacterial effect of LPCQDs was evaluated by using the standard well diffusion method in MHA plates in triplicate (*n* = 3) by following the CLSI guidelines as described before [89]. Here, *S. aureus*,* E. coli*,* S. typhi*,* P. mirabilis*, and *K. pneumoniae* were the selected pathogens with sterile distilled water used as a negative control and ciprofloxacin as a positive control. Here, volume of 60 µL LPCQDs was added into the wells made in MHA swabbed with bacterial cultures, and the zone of inhibition was measured post 24 h incubation at 37 °C [27,28].

The minimum inhibitory concentration (MIC) of LPCQDs was evaluated by the standard broth microdilution technique using sterile 96-well microtiter plates. The LCPQDs were serially diluted to obtain concentrations of 30, 15, 7.5, 3.75, 1.875, 0.937, 0.468, 0.234, 0.117, 0.058, and 0.029 mg mL^-1^ in the microwells. The MIC value of LCPQDs against each pathogen was identified based on the lowest concentration retaining blue colour visualized in the wells after the addition of indicator dye resazurin [33]. Sterile broth served as the negative control, and microbial culture without LPCQDs served as the growth control.

For the determination of minimum bactericidal concentration (MBC), 100 µL solution from the wells with no visible microbial proliferation after the incubation was spread onto sterile nutrient agar plates (*n* = 3) and incubated at 37 °C overnight. The lowest concentration of LPCQDs without any colony formation on agar media was recorded as the MBC [34].

### Antifungal activity of LPCQDs by the well diffusion method

The antifungal activity of LPCQDs was evaluated against *Candida albicans* and *Aspergillus flavus* in triplicate (*n* = 3), with amphotericin B and Nystatin as positive controls. For *C. albicans*, the inoculum was prepared with a turbidity of 0.1 OD at 600 nm and for *A. flavus*, the spore suspension was prepared as the inoculum. For the spore suspension, *A. flavus* was cultured in Potato dextrose agar (PDA) at RT until sporulation (5 to 6 days) and the mature spores were further harvested by gently scraping the surface of the media by using a sterile inoculation loop and were suspended in sterile PBS with 0.01% Tween 80. The concentration of the spore suspension was adjusted to 0.3 OD at 600 nm by using sterile PBS. The lawn culture protocol was used to inoculate the prepared fungal inoculum onto sterile PDA. Here, 0.8 mm wells were cut on the agar media by using sterile gel puncture, and 60 µL LPCQDs were added to the wells. The plates were then incubated at RT for 24 h, and for *A. flavus* until confluent growth formation. After incubation, the plates were observed for a clear zone of inhibition around the wells [35].

The Minimum Fungicidal Concentration (MFC) of LPCQDs was determined against *C. albicans* and *A. flavus* by the standard broth microdilution assay. Here, 100 µL sterile broth was added to all the wells, including the positive control, and 200 µL of sterile broth was added as the negative control. Then the LPCQDs were serially diluted to obtain different concentrations of LCPQDs. Each well was further inoculated with 100 µL of prepared inoculum. The MIC value of LCPQDs against *C. albicans* and *A. flavus* was identified based on the visual fungal growth [33].

### FE-SEM analysis for observing the Morpho-Mechanistic effect of LPCQDs

 Field Emission Scanning Electron Microscopy (FE-SEM) analysis was done by using TESCAN BRONO s.r.o. Czech, MAIA3 XMH to study the microstructure of microbial cells and to analyse the alteration in morphology upon interaction with LPCQDs. Here, cultures of *E. coli*,* S. aureus*,* C. albicans*, and *A. flavus* were incubated with LPCQDs for 4 h, and then the cultures were centrifuged at 3000 rpm (805 RCF) for 5 min at 4 ℃ to obtain the cell pellets, which were collected, and thin smears were prepared on sterile glass slides. The smears were fixed using a 2.5% gluteraldehyde solution. After drying, the prepared smear was dehydrated by treating it with 30%, 50%, 70%, and 100% ethanol. Each of the glass slides was then fixed onto the specimen holder using carbon tape and further coated with a sputtered gold film before examination [36].

### Antibiofilm activity of the synthesized LPCQDs

Here, the facile Congo red (CR) agar plate assay was adopted to screen the biofilm formation by the selected pathogenic microbial strains. The CR medium with agar (10 g L^-1^) was prepared by mixing sucrose (50 g L^-1^), CR indicator dye (0.8 g L^-1^), and the brain heart infusion Agar (BHI) (37 g L^-1^). CR solution autoclaved at 121 °C for 15 min independently from other ingredients was aseptically transferred into the autoclaved BHI media at 55 °C [28]. Prepared CRA plates were then streaked with *C. albicans*,* C. tropicalis*,* E. coli*,* S. typhi*,* P. mirabilis*,* S.aureus* and *K. pneumoniae*, and incubated for 24 h at 37 °C [37].

Quantification and comparison of the biofilm developed by *K. pneumoniae*,* S. aureus*, and *S. typhi* in the presence of LPCQDs was determined by the tissue culture plate method (TCP) mentioned in a previous report [34]. For this, four different concentrations (0, 1, 5, and 10 mg mL^-1^) of LPCQDs were used. Biofilm formed were then quantified by measuring the absorbance at 595 nm using a UV-Vis spectrophotometer [38].

Atomic Force Microscopic (AFM) analysis of the biofilm was carried out by using WITEC ALPHA300 RA - Confocal Raman Microscope with AFM to obtain the images representing the effect of LPCQDs on the biofilm of *K. pneumoniae*. Here, a 1 mL broth culture of *K. pneumoniae* was incubated with LPCQDs for 48 h in a 12-well tissue culture plate. Sterile glass slides were placed into each well, and after 48 h, the glass slides were removed from the wells using sterile forceps. Following a mild heat fixing, the smear was flooded with 2.5% glutaraldehyde. After drying, the smear was consecutively treated with 30, 50, 70, and 100% ethanol [39].

### In vitro cytotoxic effect of synthesized LPCQDs

#### Cytotoxicity assessment by MTT assay

The MTT assay was performed on L929 mouse fibroblast cells. For this, 1 mg of LPCQDs was dispersed with 1 mL DMEM and was filter sterilized using a 0.22 μm filter. After 24 h, the proliferation medium was discarded, and LPCQDs were added at dosages of 100, 50, 10, and 5 µg mL^− 1^ [40]. The LC₅₀ value for LPCQDs was calculated using nonlinear dose-response analysis. All experiments were conducted in triplicate, and results are expressed as mean values. Statistical significance relative to untreated controls was evaluated using one-way ANOVA followed by Dunnett’s post hoc test. The L929 fibroblast cell line was selected as a standard, ISO 10,993-recommended model for preliminary in vitro cytocompatibility screening of nanomaterials. Fibroblast cells are biologically relevant to the biomedical applications of LPCQDs as they are involved in tissue repair, extracellular matrix formation, and wound-healing processes. Previous reports have shown good cell viability of mouse fibroblast cell lines against citric acid functionalized carbon dots [40].

The cell viability was calculated using the formula;

$$ \% {\text{ Cell}}\;{\text{viability }} = {\text{ }}\left[ {\left( {{\mathrm{Mean}}\;{\mathrm{OD}}_{{{\mathrm{samples}}}} /{\mathrm{Mean}}\;{\mathrm{OD}}_{{{\mathrm{control}}}} } \right){\text{ x 1}}00} \right] $$

#### Cytotoxicity assay by inverted phase contrast microscopy

Here, the microtiter plate with treated cells was examined after 24 h of treatment using the microscope Olympus CKX41 and Optika Pro5 CCD Camera and the observations were documented as images. The changes detected in the appearance of treated cells, like swelling, granulation, or downsizing, and vacuole enlargement in the cells served as markers of cytotoxicity [40].

### Anti-inflammatory activity of LPCQDs

#### Bovine serum albumin (BSA) denaturation assay

For evaluating the anti-inflammatory potential of the LPCQDs, the BSA denaturation assay was performed in triplicate (*n* = 3) based on the standard methods. Here, 0.5 mL of the LPCQDs was mixed with varying concentrations (0.8, 0.6, and 0.4 mg mL^−1^) of BSA, and after an incubation period of 20 min, the solution was heated to 55 °C for 30 min in a double boiler and then cooled. Further, the absorbance at 660 nm was measured by using a spectrophotometer, and diclofenac sodium was used as a reference standard [40].

The percentage of protein denaturation (PD) was determined by using the following equation:

$$\% {\text{ PD }} = {\text{ }}\left[ {\left( {{\mathrm{Absorbance}}_{{{\mathrm{control}}}} {-}{\text{ Absorbance}}_{{{\mathrm{sample}}}} } \right)/{\mathrm{Absorbance}}_{{{\mathrm{control}}}} } \right]{\mathrm{x}}\,100. $$

#### Egg albumin denaturation assay of LPCQDs

The anti-inflammatory effects of the LPCQDs in the above-mentioned concentrations were also evaluated using the standard egg albumin denaturation assay with diclofenac sodium as the standard, and the steps were carried out as mentioned in a previous report in triplicate (*n* = 3) [41]. The PD% was determined by using the following equation:

$$ \% \;{\mathrm{of}}\;{\text{PD }} = {\text{ }}\left[ {\left\{ {({\mathrm{A}}_{{{\mathrm{control}}}} {-}{\text{ A}}_{{{\mathrm{sample}}}} )/{\text{ A}}_{{{\mathrm{control}}}} } \right\}\;{\mathrm{x}}\;{\mathrm{1}}00} \right],\;{\mathrm{where}}\;{\mathrm{A}}\;{\mathrm{is}}\;{\mathrm{the}}\;{\mathrm{absorbance}}\;{\mathrm{at}}\;{\mathrm{66}}0\;{\mathrm{nm}}.$$

The results obtained were statistically analysed using one-way ANOVA followed by Dunnett’s post hoc test in the IBM SPSS Statistics, Version 16.0, to determine significant differences at *p* < 0.05.

### Antidiabetic activity of LPCQDs

The antidiabetic effect of LPCQDs at concentrations of 1,0.8, and 0.6 mg ml^−1^ was evaluated by following the standard protocol as per the previous study [42]. The absorbance was taken at 540 nm using a multimode plate reader with quercetin, at the same dosages serving as the standard, and the controls were prepared in parallel without the test substances (LPCQDs), and each test was conducted in triplicate (*n* = 3) [39]. The percentage of enzyme inhibition (EI) was determined by the formula:

$${\mathrm{EI}}\;\% {\text{ }} = {\text{ }}\left[ {\left\{ {\left( {{\mathrm{A}}_{{{\mathrm{control}}}} - {\mathrm{A}}_{{{\mathrm{sample}}}} } \right){\text{ }}/{\text{ }}\left( {{\mathrm{A}}_{{{\mathrm{control}}}} } \right)} \right\}\;{\mathrm{x}}\;{\mathrm{1}}00} \right],\;{\mathrm{where}}\;{\mathrm{A}}\;{\mathrm{stands}}\;{\mathrm{for}}\;{\mathrm{absorbance}}\;{\mathrm{at}}\;{\mathrm{54}}0\;{\mathrm{nm}}.$$

The results obtained were statistically analysed using one-way ANOVA followed by Dunnett’s post hoc test in the IBM SPSS Statistics, Version 16.0, to determine significant differences at *p* < 0.05.

### Heavy metal ion detection

The LPCQDs were evaluated for their ability to detect the presence of heavy metals in aqueous samples. For this, 2 mL of metal salt (Mercurous nitrate, Lead sulfate, and Cadmium sulfate) was prepared in deionized water to obtain a concentration of 700 µM. Furthermore, the prepared solution was mixed with LPCQDs (1:1 v/v) at different concentrations (15, 10, 5, and 1 mg mL^-1^), and the changes in (PL) spectra were observed using a UV-Vis spectrophotometer at an excitation wavelength of 390 nm [43].

## Results

### Antibacterial activity of lemon peel extract

In the study, the antibacterial activity of the LPE was evaluated against both Gram-positive and Gram-negative bacteria. Here, LPE was observed to have significant antibacterial activity with a zone of inhibition of 20 mm against *S. aureus* and 18 mm against *E. coli* (Supplementary Fig. S1).

### Characterisation by GC-MS analysis of LPE

The GC-MS analysis of LPE prepared in this study has revealed it to have the presence of different bioactive compounds. The GC-MS chromatogram and libraries of the identified bioactive compounds, along with their relevant properties, are summarised in Table 1. Among these, 3-methylbutanoic acid had the highest percentage composition (83%) and the organic compound α-Pinene (9%) had the lowest percentage composition.

**Table 1:** Identification of phytoconstituents of lemon peel extract by GC-MS analysis

Compound name	Properties	Spectrum
3-Methylbutanoic acid	Antimicrobial, Anti-inflammatory [44]	
2,5-Furandione, 3-methyl	Antibacterial, Anti-inflammatory, ROS generation [45]	
2-Furancarboxaldehyde, 5-methyl	Antimicrobial, ROS generation [46]	
α-Pinene	Antimicrobial, Anti-inflammatory, ROS generation [47]	
o-Cymene	Antimicrobial, Anti-inflammatory, ROS generation [48]	
D-Limonene	Antimicrobial, Anti-inflammatory, ROS generation [49]	
α-Ocimene	Antibacterial, ROS generation [50]	
γ-Terpinene	Antimicrobial, Anti-inflammatory, ROS generation [51]	
5-Hydroxymethylfurfural	Antidiabetic, antibiofilm [52]	
cis-α-Farnesene	Antimicrobial [53]	
cis-β-Bisabolene	Antibacterial, ROS generation [54]	
Melezitose	Antidiabetic [55]	

### Characterization of LPCQDs

**Fig. 1:**
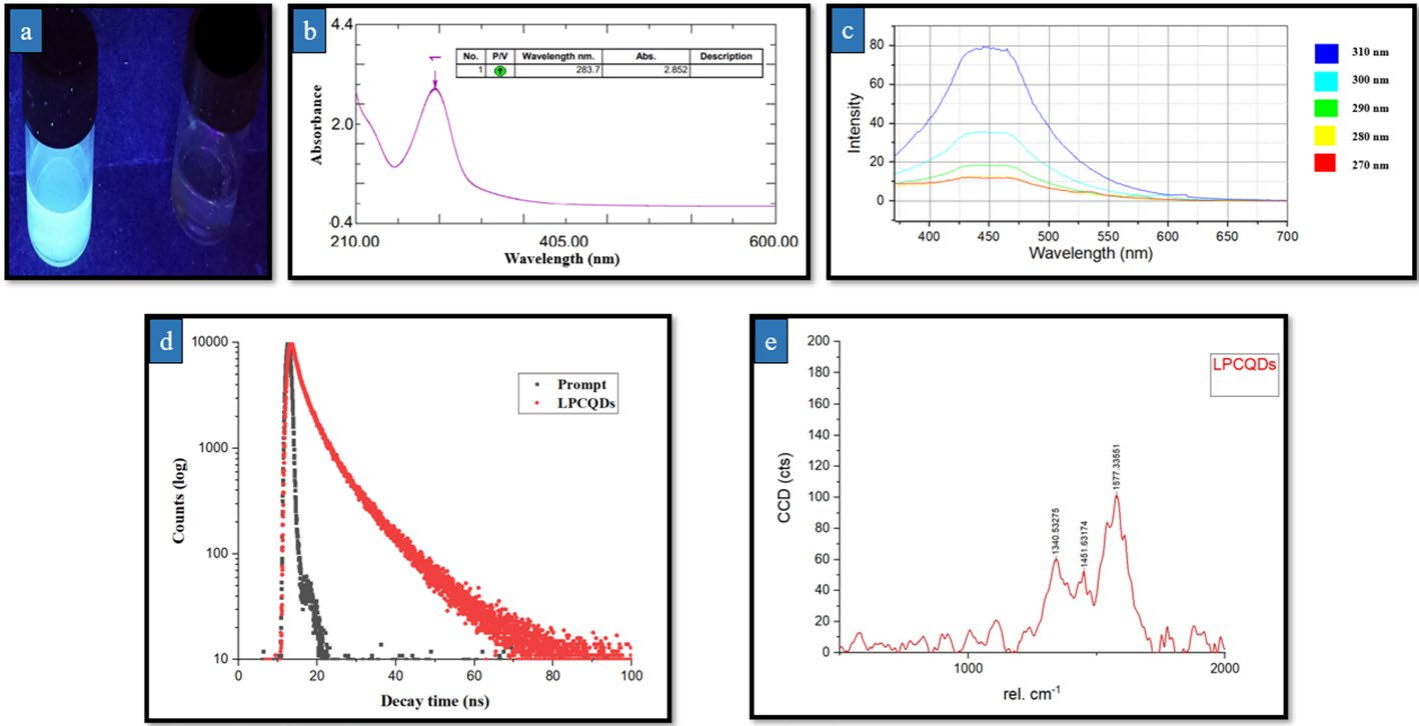
Characterisation of the synthesized LPCQDs: **a** blue light emission by the LPCQDs under UV irradiation,** b** UV-Vis absorption spectrum of the LPCQDs,** c** fluorescence emission spectrum of the synthesized LPCQDs,** d** fluorescence lifetime decay of LPCQDs, and** e** Raman spectrum of LPCQDs synthesized in the study

In the study, A concentration of 30 mg mL^-1^ was achieved, and a total volume of 20 mL LPCQDs was obtained from 20 mL of ethanolic lemon peel extract, corresponding to an overall CQDs yield of approximately 600 mg. Based on the final concentration and volume of the obtained dispersion, approximately 600 mg of lemon peel-derived carbon quantum dots (LPCQDs) were produced from 6 g of dried lemon peel precursor, corresponding to an overall yield of ~ 10% mass conversion efficiency. In the present study, the formation of QDs was initially confirmed by the blue fluorescence observed upon UV light exposure (Fig. 1a).

The optical properties of the LPCQDs were studied by using a UV-Vis spectrophotometer (Fig. 1b). Here, the LPCQDs synthesised showed a prominent absorption peak at 283 nm in the UV region. This could be mainly attributed to the n-π* transitions occurring due to the C = C molecular bonds. The solution also exhibited a bandgap corresponding to the blue region of the visible spectrum, indicating the successful synthesis of LPCQDs. The series of emission spectra of the LPCQDs solution at different excitation wavelengths (270, 280, 290, 300, and 310 nm) has been demonstrated in Fig. 1c. The intensity of the emission peak here was found to increase with the rise of the excitation wavelength from 270 to 310 nm, and at the excitation wavelength of 310 nm, the emission intensity was found to reach the maximum. The PL studies have revealed the mean PL intensity of LPCQDs at 30 mg mL^− 1^ to be 68.48 ± 6.7 a.u. and at 15 mg mL^− 1^ to be 29.48 ± 1.49 a.u., after 12 months storage in dark refrigerated conditions.

The Quantum yield (QY) of the synthesized LPCQDs, when excited at 350 nm along with the maximum emission at 450 nm, was calculated to be 7.4%, which indicated its moderate fluorescence efficiency. The average fluorescence lifetime of the LPCQDs obtained in the present study was 4.48 ns. Figure 1(d) could demonstrate the fluorescence lifetime decay of the synthesized LPCQDs when compared to the prompt or reference sample. Here, the prompt decay was observed to be faster than the LPCQDs, which indicated a shorter fluorescence lifetime. The LPCQDs were found to have a slower decay, indicating a longer fluorescence lifetime when compared to the prompt. The Raman spectrum analysis of LPCQDs has displayed two broad peaks at 1350 and 1577 cm^− 1^ representing sp^3^-hybridized disordered D and sp^2^ - hybridized graphitic G bands, respectively, with an I_D_/I_G_ band intensity of 0.75 Fig. 1(e).

**Fig. 2:**
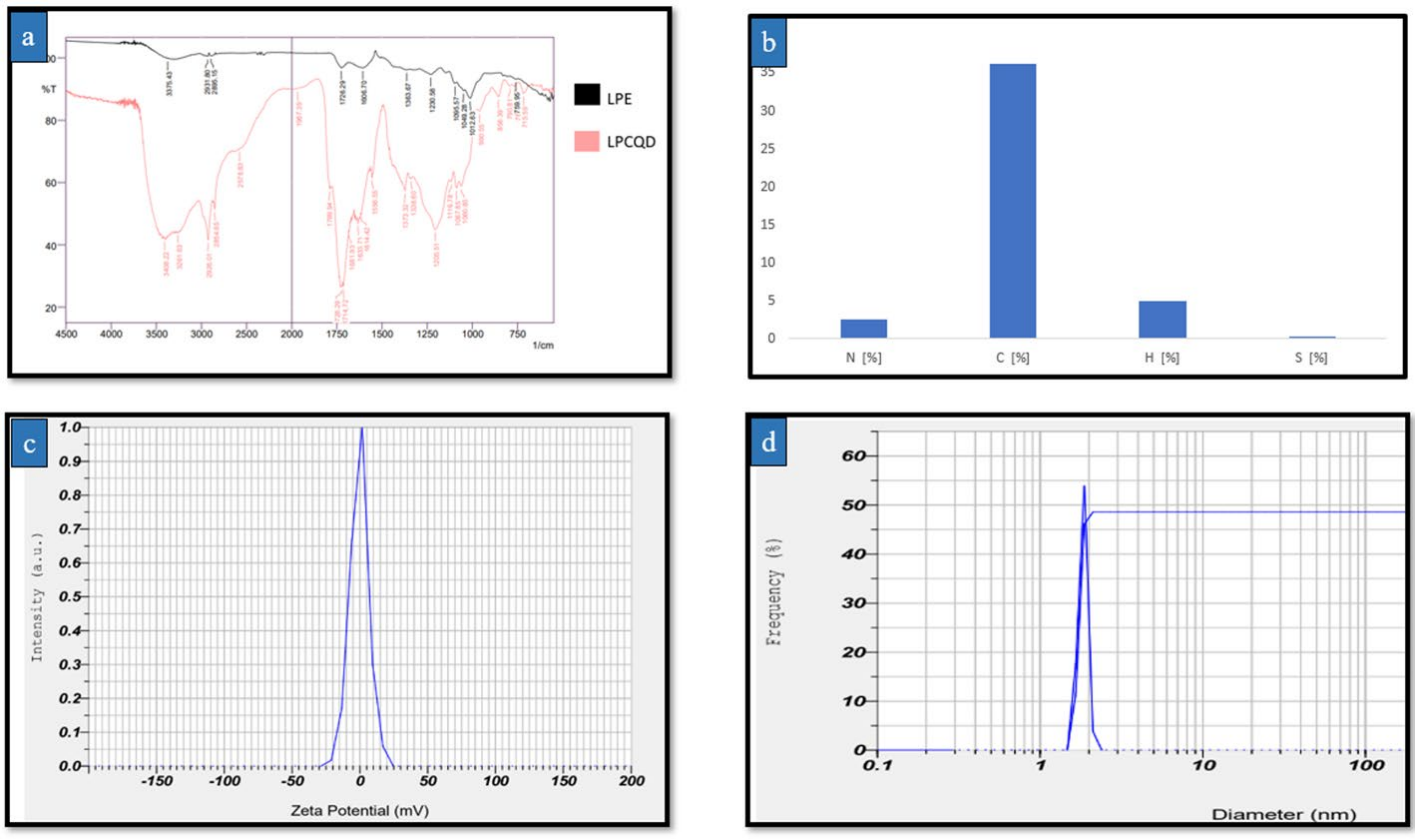
Structural and compositional analysis of LPCQDs: **a** FT-IR spectrum of LPCQDs along with LPE, **b** CHNS analysis of LPCQDs synthesized in the study, **c** zeta potential analysis of the synthesized LPCQDs, and **d** DLS of LPCQDs

In the study, the FT-IR spectroscopic analysis of both the LPCQDs and LPE was conducted to study and compare the functional groups. Here, the FT-IR spectra of both the LPCQDs and LPE were found to have a comparable distribution of functional groups. The FT-IR spectrum of LPE has shown the presence of broad peaks around 3400 cm^− 1^ corresponding to the O-H stretching vibrations of hydroxyl groups of compounds like sugars and polyphenols. Peaks around 2900 cm^− 1^ may be attributed to the C-H stretching vibrations from the hydrocarbon chains. Peaks around 1600 cm^− 1^ corresponded to the C = O stretching of carbonyl groups, likely from compounds like citric acid and flavonoids. Peaks formed around 1400 cm^− 1^ could be due to the C-O-H bending vibrations. The FT-IR spectrum of LPCQDs showed a shift and sharpening of the O-H stretching peak around 3400 cm^− 1^ when compared to the LPE. New peaks appeared around 1700 cm^− 1^, which correspond to the C = O stretching of carboxyl groups, indicating the formation of carboxylated CQDs. Peaks around 1600 cm^− 1^ became more pronounced, suggesting an increase in C = C bonds in the CQDs structure. Peaks around 1400 cm^− 1^ were also more defined, corresponding to the C-O-H and C-O stretching (Fig. 2a).

According to the results of CHNS analysis, the LPCQDs synthesized in the study were found to contain 36% carbon, 2.51% nitrogen, 4.85% hydrogen, and 0.059% sulfur (Fig. 2b). The absolute value for the zeta potential of LPCQDs synthesized in this study was observed to be -0.5mV (Fig. 2c). The observed negative value for the zeta potential could be an indication of repulsive electrostatic forces between the molecules. As the observed zeta potential of LPCQDs was - 0.5 mV, it could be considered to have an anionic nature. Here, the particle size distribution of LPCQDs was determined by using the DLS, as shown in Fig. 2(d). The results obtained here indicate the particles have sizes that range from 1.7 to 2 nm, which could also be confirmed by the TEM analysis. The small particle size implies the presence of a higher surface area to volume ratio, resulting in more reactive particles.

**Fig. 3:**
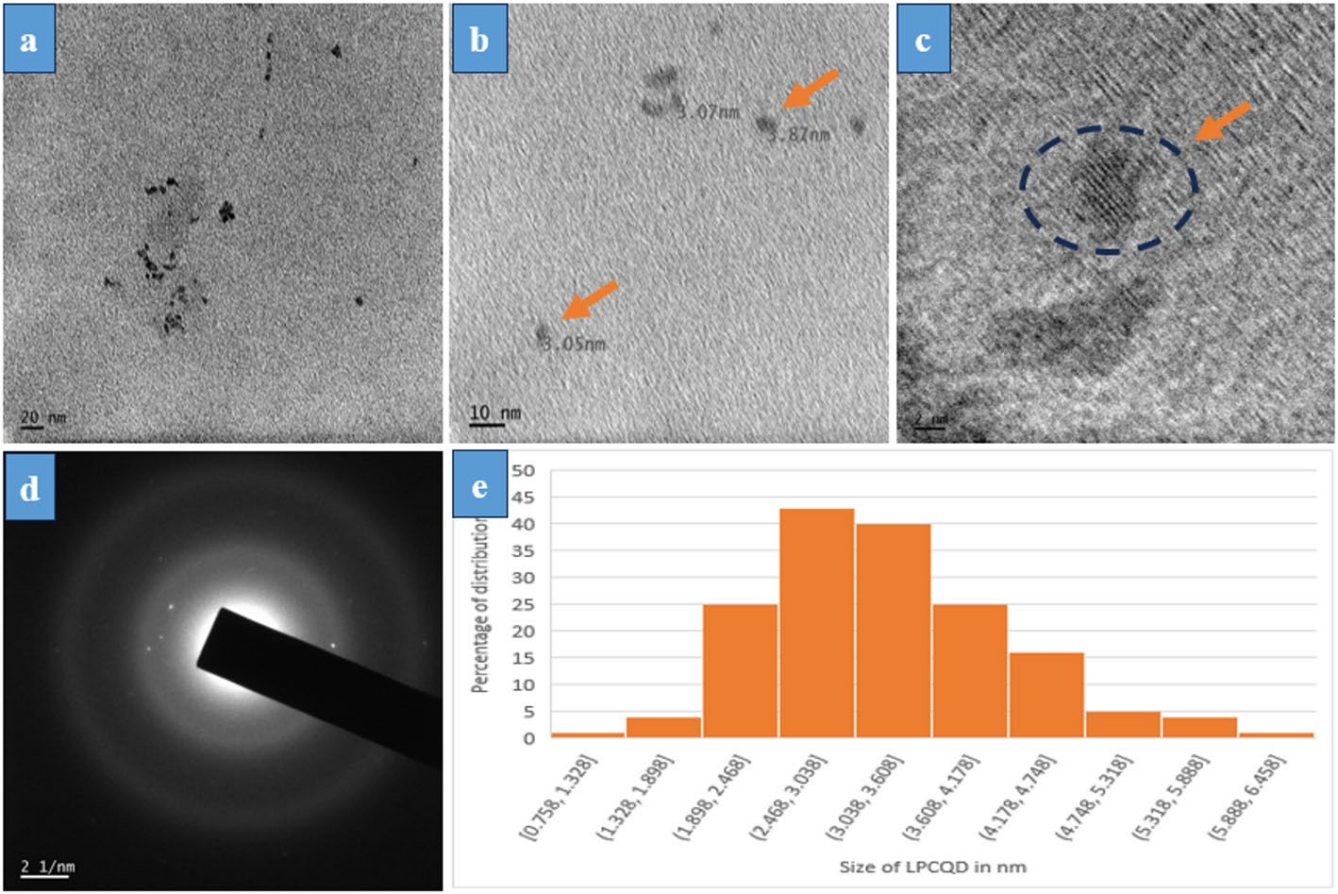
TEM analysis of LPCQDs at different magnifications: **a** 20 nm, **b** 10 nm scale, and **c** 2 nm **d** SAED pattern and **e** particle size histogram analysis of LPCQDs

The TEM analysis of LPCQDs at various magnifications has confirmed that they have a quasispherical shape with monodisperse structures well separated from each other. The size distribution of LPCQDs was found to be in the range of 2-4 nm (Fig. 3a, b and c). Figure 3(e) illustrates the particle size histogram analysis of LPCQDs synthesized in the study and analysed by the TEM images. Here, the average size of LPCQDs was determined to be 2.75 nm.

### Antibacterial activity of LPCQDs

**Fig. 4:**
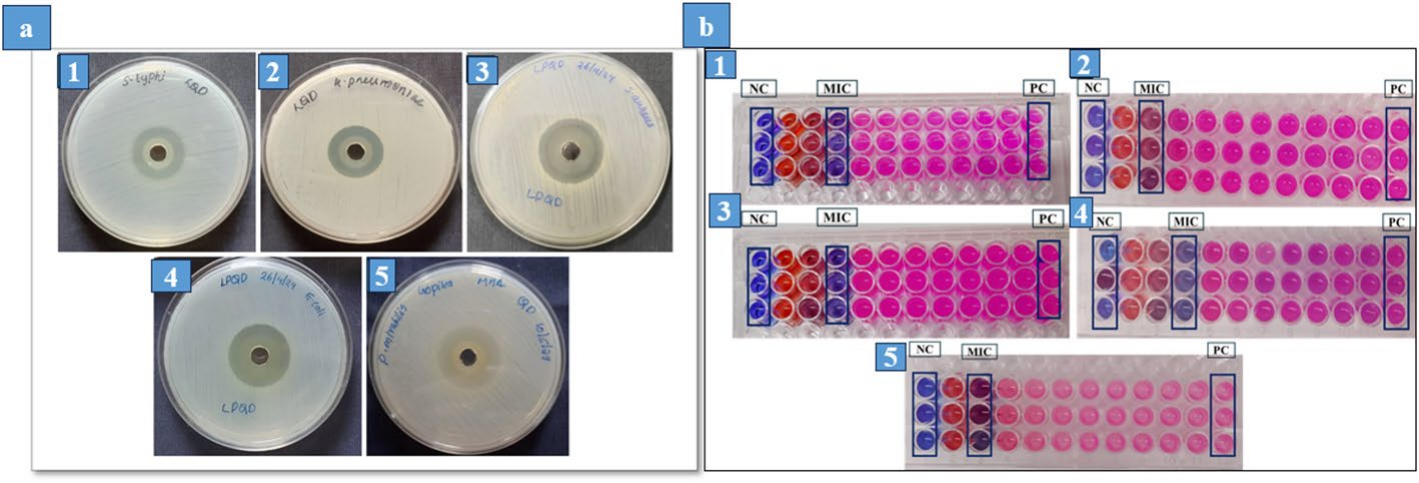
Antibacterial activity analysis: (**a**) agar well diffusion and (**b**) MIC of LPCQDs against (1) *S. typhi*, (2) *K. pneumoniae*, (3) *S. aureus*, (4) *E. coli*, and (5) *P. mirabilis*

The antibacterial activity analysis of LPCQDs was carried out by using the agar well diffusion method. Here, the zone of inhibition observed for LPCQDs was 28 ± 0 mm against *S. aureus*, 26 ± 0 mm against *E. coli*, 22 ± 0 mm against *S. typhi*, 25 ± 0 mm against *K. pneumoniae* and 26 ± 0 mm against *P. mirabilis* respectively (Fig. 4a). The MIC of LPCQDs against *E. coli*,* S. aureus* and *S. typhi* was observed to be 0.94 ± 0 mg mL^− 1^ and for *K. pneumoniae* and *P. mirabilis* it was 1.88 ± 0 mg mL^− 1^ (Fig. 4b). The MBC of LPCQDs against *E. coli*,* S. aureus*,* S. typhi*,* K. pneumoniae*, and *P. mirabilis* was found to be 3.75 ± 0 mg mL^− 1^. From these, a comparison was made of the MIC and MBC values of LPCQDs as mentioned in Table S1 of supplementary data.

### Antifungal activity of LPCQDs

**Fig. 5:**
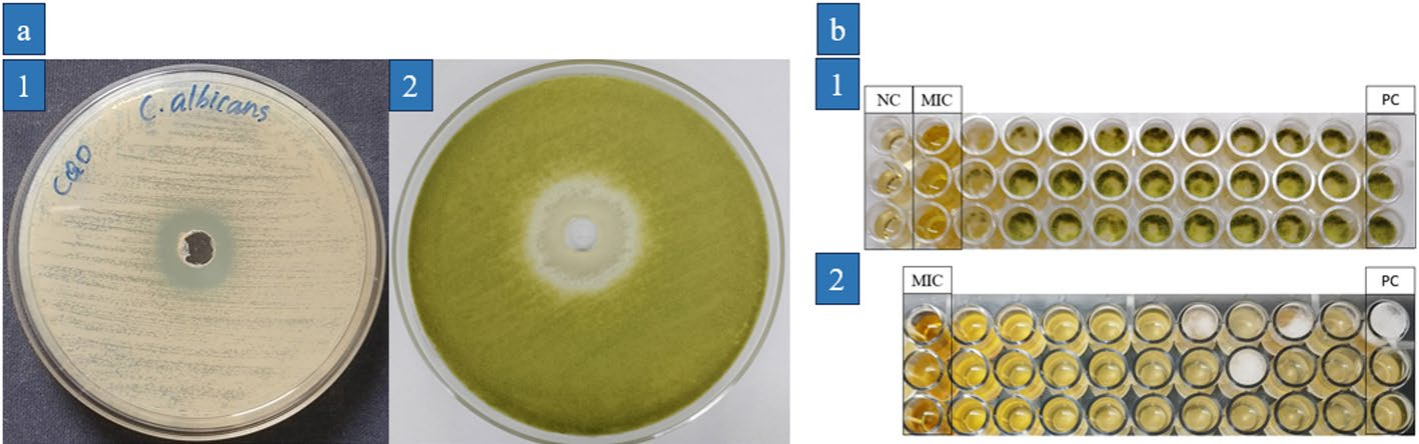
Antifungal activity of LPCQDs: (**a**) antifungal activity analysis by well diffusion and (**b**) MFC of LPCQDs against (1) *C. albicans* and (2) *A. flavus*

The antifungal activity of LPCQDs was also carried out by using the standard agar well diffusion method against standard strains. Here, the zone of inhibition for a volume of 60 µL of LPCQDs was found to be 17 ± 0 mm against *C. albicans*, and 15 ± 0 mm against *A. flavus* (Fig. 5a). The MFC of LPCQDs was further determined by using the standard 96-well microtiter plate method. Here, the MFC values of LPCQDs against both *C. albicans* and *A. flavus* were determined to be 3.75 ± 0 mg mL^− 1^(Fig. 5b).

### The Morpho-mechanistic effect of LPCQDs on microorganisms

**Fig. 6:**
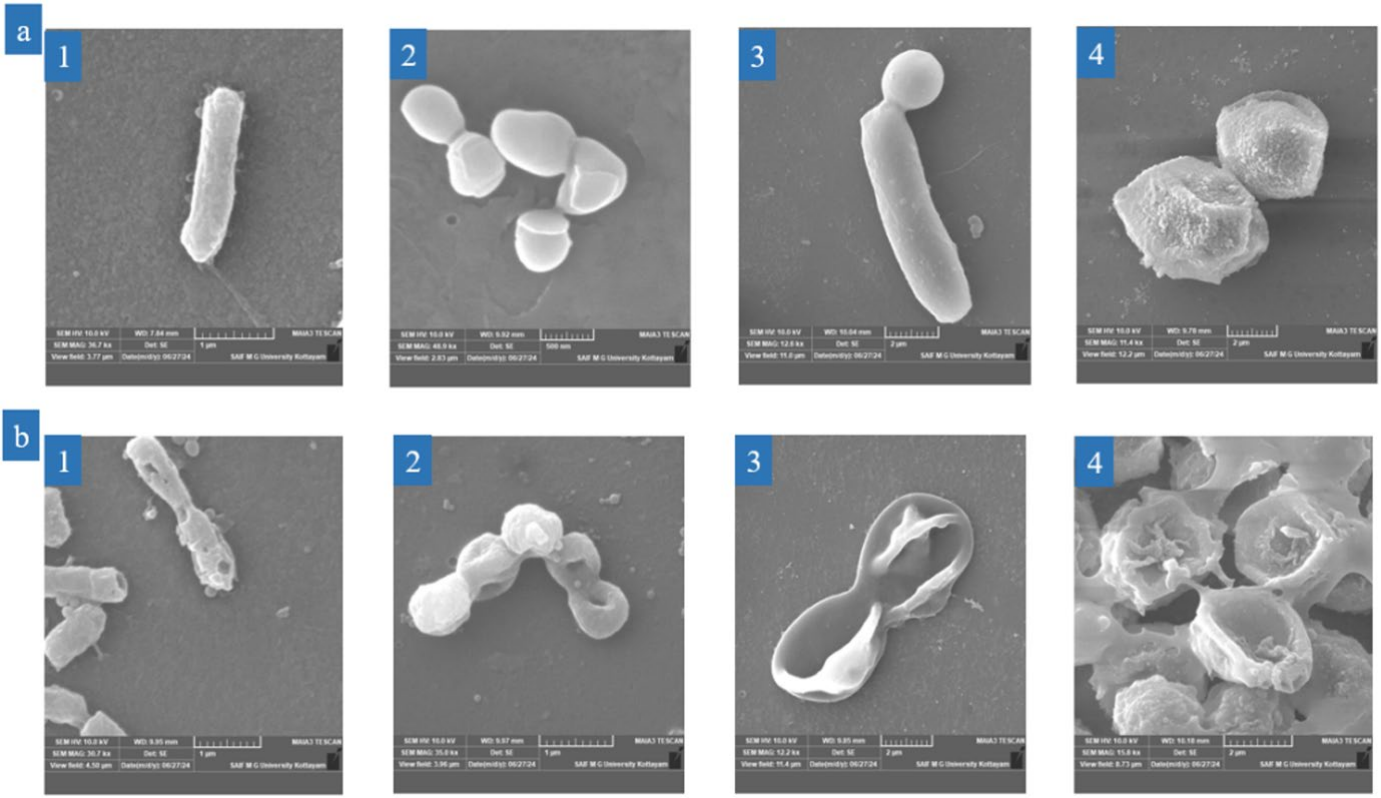
FE-SEM analysis of (**a**) untreated and (**b**) LPCQDs-treated microbial cells (1) *E. coli*, (2) *S. aureus*, (3) *C. albicans*, and (4) *A. flavus*, respectively

Here, the FE-SEM analysis of microbial cells treated with LPCQDs was carried out to compare the surface morphology of selected microorganisms. Here, the untreated *S. aureus*,* E. coli*,* C. albicans*, and *A. flavus* were found to retain their morphological integrity with clear and smooth edges. On the contrary, cells treated with LPCQDs were found to have a disrupted cell membrane (Fig. 6).

### In-vitro cytotoxic effect of synthesized LPCQDs

**Fig. 7:**
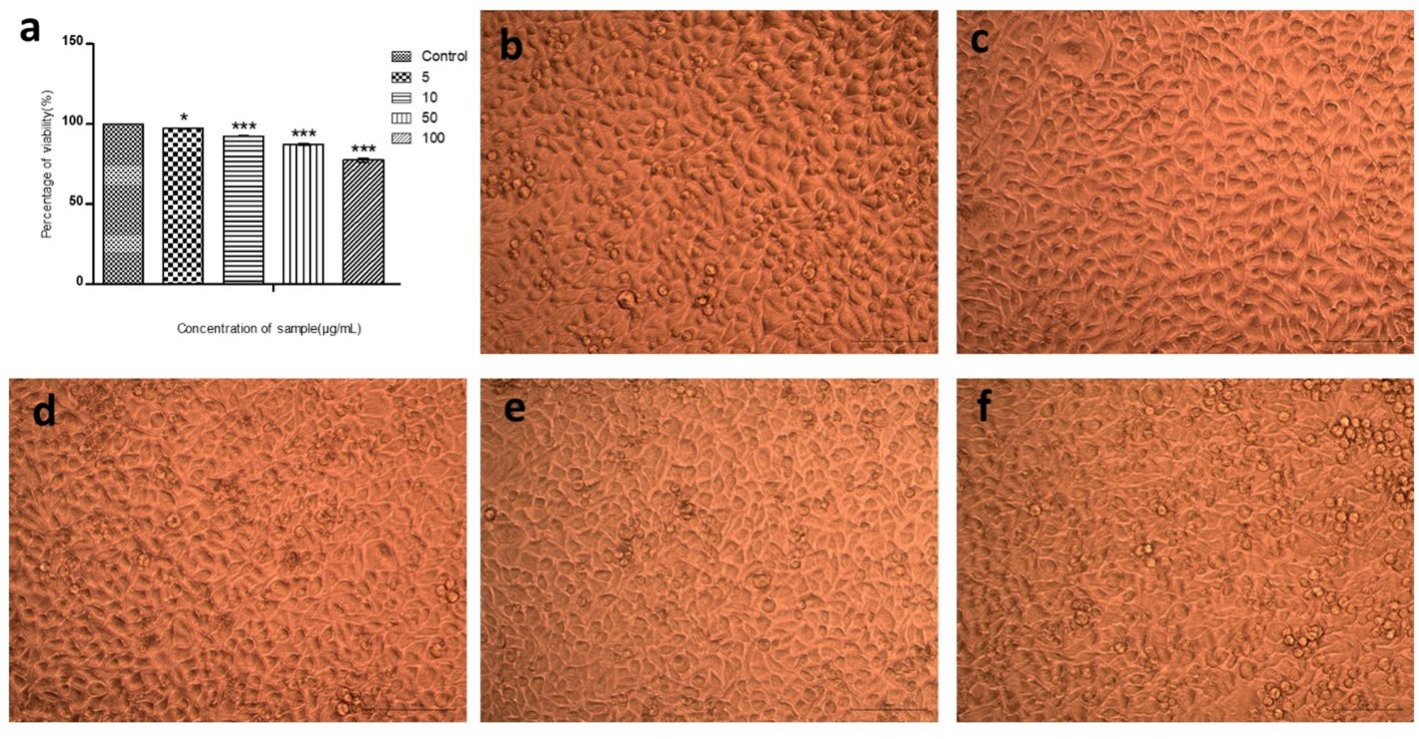
Cytotoxicity analysis of LPCQDs: (**a**) Graphical representation illustrating the cell viability of L929 cells at different treatments and cell viability observed for the different concentrations of LPCQDs in L929 fibroblast cell lines (**b**) Control (untreated), (**c**) treated with 5 µg mL^− 1^ LPCQDs, (**d**) 10 µg mL^− 1^ LPCQDs, (**e**) 50 µg mL^− 1^ LPCQDs, and (**f**) 100 µg mL^− 1^ LPCQDs

To determine the biocompatibility of LPCQDs, the standard MTT assay using the L929 (Mouse Fibroblast) cell line was carried out. The viability of treated cells was found to be ≈ 97% at the lowest concentration (5 µg mL^− 1^) tested and ≈ 92% at 10 µg mL^− 1^. Also, 87.29 and 77.51% viability was observed for 50 and 100 µg mL^− 1^ treatments, respectively. Here, the LC50 value of LPCQDs was calculated to be 45.667 µg mL^− 1^at 24 h exposure (Fig. 7).

### Antibiofilm activity of LPCQDs

**Fig. 8:**
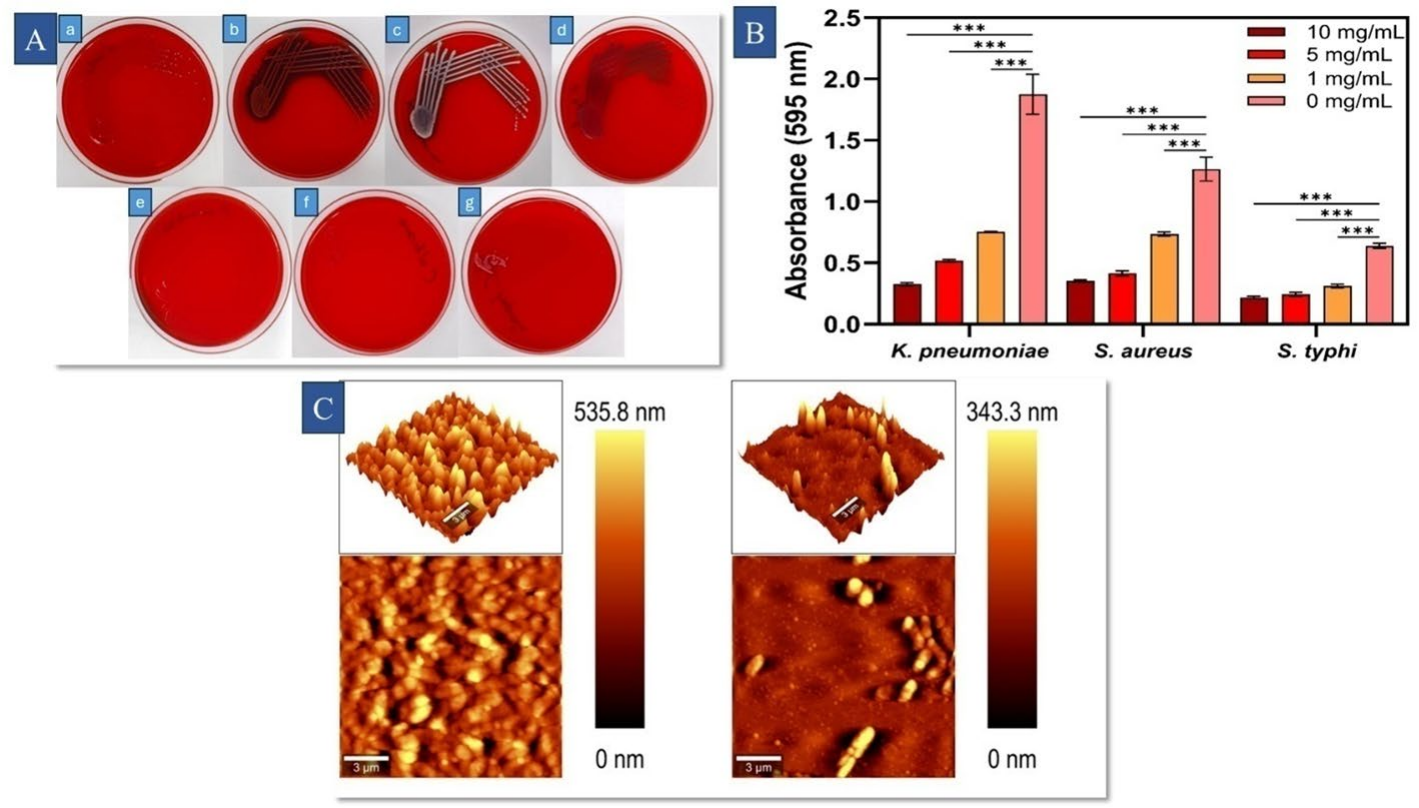
Biofilm formation by selected microbial strains and antibiofilm activity of LPCQDs (**A**) Congo red assay-based screening of biofilm formation in (**a**) *E. coli*, (**b**) *S. aureus*, (**c**) *K. pneumoniae*, (**d**) *S. typhi*, (**e**) *P. mirabilis*, (**f**) *C. albicans*, and (**g**) *C.tropicalis*; (**B**) Bar diagram representing the antibiofilm activity of LPCQDs against *K. pneumoniae*,* S. aureus*, and *S. typhi* at four different concentrations. (**C**) AFM images of antibiofilm activity of LPCQDs against *K. pneumoniae*: Untreated control and LPCQDs-treated biofilm

In the Congo red assay, *K. pneumoniae* was found to produce black colonies, hence categorized as strong biofilm-forming bacteria, and *S. aureus* and *S. typhi* were observed to be moderate biofilm-forming bacteria. The antibiofilm activity analysis of LPCQDs was analyzed against the biofilm-forming *K. pneumoniae*,* S. aureus*, and *S. typhi*. Here, the LPCQDs were observed to demonstrate significant antibiofilm activity. Here, treatment with LPCQDs showed 82.5 ± 0.6, 72.1 ± 0.7 and 65.9 ± 1.7% of biofilm inhibition for *K. pneumoniae*,* S. aureus*, and *S. typhi*, respectively. The AFM analysis of the biofilms formed by treated and untreated *K. pneumoniae* (Fig. 8) also demonstrated the antibiofilm activity of LPCQDs. Surface topography images of untreated samples showed dense and thick biofilm, whereas the samples treated with LPCQDs showed the absence of a continuous biofilm. This indicated the LPCQDs to have an impact on the structural composition and integrity of the biofilm formed by *K. pneumoniae.*

### **Anti-inflammatory activity of LPCQDs**

**Fig. 9:**
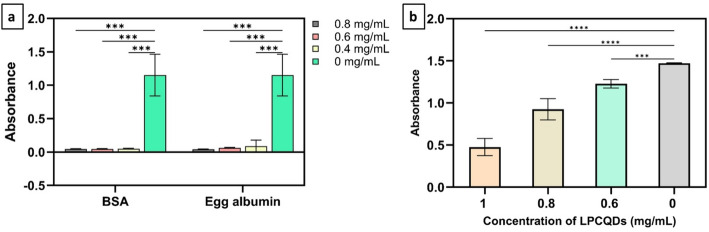
Bioactivity of LPCQDs: (**a**) anti-inflammatory activity of LPCQDs synthesized in the study, and (**b**) antidiabetic activity of LPCQDs, *p* < 0.05

Here, both the egg albumin assay and the bovine serum albumin assay results demonstrated a significant concentration-dependent inhibition of protein denaturation at 0.8, 0.6, and 0.4 mg mL^− 1^ concentrations of LPCQDs. The higher the concentration of LPCQDs, the lower the protein denaturation observed. At 0.8 mg mL^-1^, LPCQDs showed 96.01 ± 1.64% inhibition of BSA denaturation, which was statistically comparable to diclofenac sodium (96.21 ± 0.31%). In the egg albumin assay, LPCQDs demonstrated 79.29 ± 0.53% inhibition, while diclofenac exhibited 96.7 ± 0.36% inhibition, indicating slightly reduced efficacy likely due to the rigid disulfide-rich structure of egg albumin. The results hence revealed the LPCQDs to have potent anti-inflammatory activity, which was comparable with that of standard diclofenac sodium (Fig. 9a) (Table 2).

**Table 2:** Anti-inflammatory activity of LPCQDs

LPCQDs (mg mL^− 1^)	0.8	0.6	0.4
% Inhibition of BSA denaturation	96.01 ± 1.64	95.85 ± 2.81	95.69 ± 2.29
% Inhibition of egg albumin denaturation	79.29 ± 0.53	72.49 ± 0.47	58.34 ± 0.58
*Diclofenac standard (mg mL* ^*− 1*^ *)*	**0.8**	**0.6**	**0.4**
% Inhibition of BSA denaturation	96.21 ± 0.31	96.08 ± 0.13	95.41 ± 0.37
% Inhibition of Egg albumin denaturation	96.7 ± 0.36	95.61 ± 0.21	94.95 ± 0.36

### Antidiabetic activity of LPCQDs

Here, the LPCQDs showed antidiabetic activity by inhibiting the reaction of α-amylase. The obtained results indicate the inhibition of amylase increased with the LPCQDs concentration, reaching a 65.8 ± 0.1% inhibition at 1 mg mL^− 1^,39.56 ± 0.03% at 0.8 mg mL^− 1^ and 16.44 ± 0.05% at 0.6 mg mL^− 1^. This trend suggests a dose-dependent inhibition by LPCQDs, indicating that LPCQDs show significant suppression (*p* < 0.05) of activity of α-amylase(Fig. 9b) (Table 3).

**Table 3:** %Inhibition of α-amylase

	1 mg mL^− 1^	0.8 mg mL^− 1^	0.6 mg mL^− 1^
LPCQDs	65.8 ± 5.85	39.56 ± 6.97	16.44 ± 3.78
Quercetin	64.30 ± 0.77	61.83 ± 0.93	56.24 ± 0.82

### Heavy metal ion detection by LPCQDs

**Fig. 10:**
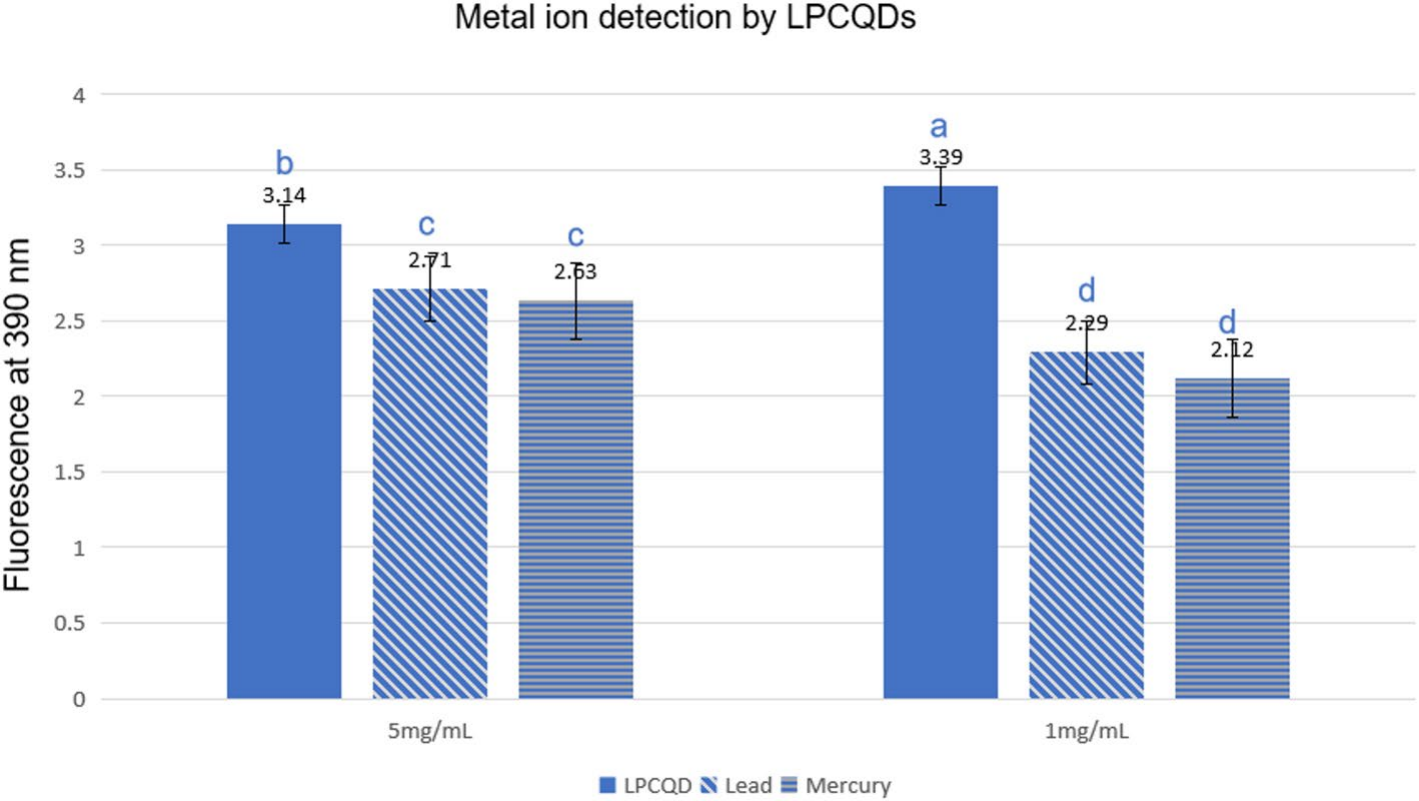
Fluorescence of mercury and lead ions at two different concentrations in the presence of LPCQDs

To evaluate the efficiency of LPCQDs for detecting mercury and lead ions, PL based analysis was carried out at RT. Here, two different concentrations of LPCQDs (5 and 1 mg mL^− 1^) were treated with mercury and lead ions at a concentration of 700 µM. The PL was further measured at 390 nm, and from the results, “turn-off fluorescence” could be observed for both the mercury and lead ions in the presence of LPCQDs (Fig. 10). The limit of detection (LOD) for Hg²⁺ and Pb²⁺ sensing by the synthesized carbon quantum dots (CQDs) was estimated according to the equation **LOD = 3σ/S**, where σ represents the standard deviation of the blank signal (CQDs in the absence of metal ions) and S denotes the sensitivity of the sensor. The blank standard deviation was obtained from three independent measurements of CQDs without metal ions. Sensitivity was estimated by assuming a linear response between 0 and 700 µM metal ion concentration, using the change in optical response observed upon addition of 700 µM Hg²⁺ or Pb²⁺. Based on this approach, the estimated LOD for Hg²⁺ sensing using CQDs (1 mg) was approximately **26.99 µM**, and for Pb²⁺ detection using CQDs (1 mg) was calculated to be approximately **31.17 µM**, indicating effective metal-ion-induced signal modulation.

## Discussion

The synthesized LPCQDs were very versatile due to their high carbon content and surface active functional groups as evidenced in the CHNS and FTIR analysis. Compared to other carbon dots, LPCQDs were exceptionally stable even after storing in dark, low temperature conditions for more than 12 months, as demonstrated in our PL studies. The study overcomes some of the major disadvantages in the conventional process of synthesizing CQDs, including the use of corrosive chemicals, high energy consuming tools, the use of non-renewable resources and lengthy, complicated protocols.

Compared to previous studies on LPCQDs, the present study also extends the carbon dots research to the highly relevant biological properties like antibiofilm, anti-inflammatory, and anti-diabetic potential of the carbon dots. Early studies on LPCQDs used the hydrothermal synthesis at higher temperatures of 200℃ for extended time periods, resulting in fluorescent carbon dots; however, these synthesis methods were either accompanied by multiple pretreatment steps like drying at high temperatures and hazardous chemical reagents [8], which are laborious and time consuming. Where fewer pretreatment steps were involved, the synthesis resulted in CQDs with larger morphological sizes [56]. Some studies employed microwave assisted pyrolytic methods, which were time saving as compared to the hydrothermal processes, but still had multiple synthesis steps to achieve comparable results [57]. A study in 2023 reported carbon dots from lemon bagasse using microwave pyrolysis and resulted in carbon dots with low cytotoxicity compared to the present study, but this investigation was limited to the study of antioxidant potential and nanothermometric applications of carbon dots [58]. Another previous report used a simple hot water extract from lemon peel wastes, followed by microwave irradiation, but resulted in a very low quantum yield of LPCQDs (0.81%) with no metal sensing or bioactive properties [15]. A recent study employed the hydrothermal method at a higher temperature of 200℃ for a longer time period of 12 h, but still had several steps using methanol, sulphuric acid and sodium hypochlorite along with multiple rinsing steps utilising large amounts of deionised water. This is chemically and energetically intensive and can contradict the very spirit of sustainability of the method [59]. Hence, a balance in the choice of reagents and the steps involved, resulting in multifunctional LPCQDs, would be highly desirable to achieve sustainability goals and scientific advancement in this field, concurrently. Table S2 provides a comparison of the results of the current study along with the previous literature on LPCQDs.

Based on the GC-MS analysis, the LPE prepared in this study was found to be abundant with various bioactive compounds containing the reactive functional groups. Compounds like α-pinene, O-cymene, D-limonene, and γ-terpinene in lemon peel have been recognized for their antimicrobial effects. Lemon peel also contains compounds like 3-methylbutanoic acid, in addition toα-pinene, and o-cymene, all of which are known to have anti-inflammatory activity and are hence used in medical applications for reducing inflammation-related conditions. Other compounds in lemon peel, such as 2,5-furandione and α-pinene, have already been known to have functional groups with reactive oxygen species (ROS) generation potential, which could be replicated in the LPCQDs in the coated form. The LPCQDs’ ability to generate ROS could be beneficial for bringing about the antibacterial activity by causing oxidative stress in bacterial cells. When compared to the QDs reported in previous studies, the LPCQDs synthesized from lemon peel may form a sustainable and bio-friendly alternative to conventional antimicrobial materials. This aligns with the findings by *Molaei et al.* [60], and *Parvathy et al.* [61], where the plant-derived CQDs have been reported to retain their bioactivity due to the functional groups derived from the original biomass. CQDs derived from the lemon peel might have the inherent ability to inhibit enzymes involved in carbohydrate metabolism, like α-amylase and α-glucosidase. This property makes the LPCQDs synthesized in this study to be a potential antidiabetic agent to regulate the blood sugar levels.

The formation of LPCQDs in this study was initially identified by the UV-Vis spectroscopy, where an absorption peak of 283 nm could be observed, and this was in agreement with the previous report, which demonstrated the same between 200 and 300 nm [25]. Ethanol removal at 60 °C primarily serves to eliminate residual solvent without inducing additional carbonization or structural modifications. Moreover, oxygen-containing surface functional groups responsible for fluorescence emission are generally stable under these mild conditions. Hence, ethanol removal does not affect the properties or composition of the carbon dots. The fluorescence emission spectrum of LPCQDs observed in the study showed that the intensity of the emission peak increased with the rise of the excitation wavelength from 270 to 310 nm, and at 310 nm, the emission intensity was observed to reach the maximum. As the LPCQDs were found to exhibit excitation-dependent PL, the emission wavelength shift could also be expected to be based on the used excitation wavelength. The peak obtained at 450 nm indicated a specific excitation condition that optimizes the fluorescence output, which is an essential factor for calibrating sensors and enhancing the sensitivity of detection [62]. Additionally, PL studies were conducted, and the LPCQDs were found to retain fluorescence as evidenced by the observed mean PL intensity of 68.48 ± 6.7 a.u. even after 12-month storage in airtight, dark conditions at low temperatures. It has been previously reported that carbon dots can remain photostable for long time-periods when stored under conditions protected from UV light and lower temperatures [63]. Previous reports on the stability of LPCQDs have proven minimal change in PL intensity of carbon dots in normal conditions for up to 55 days when stored in refrigerated conditions [57].The retention of PL peaks at an excitation wavelength of 310 nm in the stored samples also confirms the optical stability of the LPCQDs dispersion. Although zeta potential is -0.5 mV, indicating low electrostatic stability, it is noteworthy that the retention of PL indicates comparably stable dispersion, which may be due to the spatial stabilising action of the surface active functional groups like hydrogen bonds inherited from the phenols and flavonoids in the lemon peel [64]. Hence, it can be concluded that here, the steric stabilisation overcomes the lack of electrostatic repulsion, thereby avoiding self-quenching and aggregation as well as making the dispersion stable despite the zeta potential being - 0.5 mV.

The quantum yield (QY) of LPCQDs when excited at 350 nm with the maximum emission at 450 nm, was observed to be 7.4%, which indicated its moderate fluorescence efficiency. The CQDs synthesized from watermelon fruit peels have previously demonstrated a QY of around 7.1% [65]. Moderate QYs are typically associated with the CQDs synthesized without extensive doping or surface engineering, often resulting from greener synthesis methods. Although a previous report [66] has shown the successful synthesis of good quality carbon dots even at a lower temperature of 180 ℃ during the hydrothermal treatment, the lower temperatures may result in moderate quantum yields due to the lesser extent of carbonization [67].

The average fluorescence lifetime of the LPCQDs obtained in this study was 4.48 ns, and the observation is relatively moderate when compared to the previous reports. This could be due to several factors, such as the size and surface chemistry of the formed CQDs, the excitation wavelength used, and the presence of quenching agents. Previous studies have already reported the fluorescence lifetime for the CQDs to be in the range from a few nanoseconds to several hundred nanoseconds. For example, Zhou et al. [65] have previously reported a fluorescence lifetime of nearly 5.7 ns for the CQDs synthesized using a hydrothermal method from watermelon. At the same time, Wang et al. [68] have previously reported a fluorescence lifetime of 220 ns for the CQDs synthesized using the microwave plasma method. The shorter fluorescence lifetime of LPCQDs observed in this study could be due to its smaller size, which favours faster nonradiative decay. The surface chemistry of the LPCQDs could also play a role due to the presence of surface functional groups, which could quench the fluorescence. Additionally, the excitation wavelength used in this study (450 nm) might be closer to the absorption band of the CQDs, which could also lead to faster nonradiative decay.

The Raman spectrum of the LPCQDs synthesized in the study displayed two broad peaks at 1350 and 1577 cm^-1^ representing the D (sp^3^-hybridized disordered D) and G (sp^2^-hybridised graphitic G) bands, respectively, with an I_D_/I_G_ band intensity of 0.75. The observed I_D_/I_G_ ratio is less than 1, indicating a moderate level of structural disorder in the synthesized LPCQDs. This could be due to the balance between the defective (amorphous) carbon and more ordered graphitic domains typical for the CQDs. Previous studies have also reported the varying ID/IG ratio for the CQDs, due to the synthesis method and the source material used. For example, CQDs synthesized from glucose have been reported to have an ID/IG ratio ranging from 0.5 to 1.0. whereas the CQDs from carbon nanotubes had values ranging from 0.2 to 0.8 [69]. The ID/IG ratio of 0.75 is also indicative of structural defects in the carbon dots, like edge sites, vacancies, and disruptions linked to heteroatoms in the core structure. Previous reports have elucidated that such features help in the generation of reactive oxygen species through local bandgap states under physiological conditions [70]. The CQDs accomplish their antimicrobial properties through multiple mechanisms, and one of the primary mechanisms is ROS generation. When these vacancies and defects are present, they trigger the production of ROS when exposed to the cellular environment. The ROS produced in the cytoplasm oxidise intracellular targets like proteins, lipids, and nucleic acids with ultimate microbial death [71].

FTIR analysis has been used in this study to identify the functional groups of LPCQDs, which showed the characteristic peaks at 3408 cm^− 1^, 2926 cm^− 1^, 1726 cm^− 1^, and 1250 cm^− 1^. The peak at 3408 cm^− 1^ is characteristic of O–H stretching vibrations, commonly associated with the adsorbed water on the LPCQDs surface. Previous reports have demonstrated the retention of functional groups from the precursor orange peel phytochemicals in the synthesized CQDs, and similar results were observed in the FTIR results of the current study [72]. The presence of hydroxyl groups is common in CQDs synthesized from organic precursors, and they introduce surface polarity, improve solubility in aqueous media, and enable hydrogen bonding, which is useful in bioimaging and sensing applications [73]. The peak formed at 2926 cm^− 1^ was indicative of C–H stretching vibrations, which might have originated from the aliphatic –CH₂ or –CH₃ groups of the carbonaceous precursors or capping agents used during the CQDs synthesis. Aliphatic C–H groups might have contributed to the hydrophobic properties on the CQDs surface, which can influence the CQDs stability and interaction with other molecules [74]. The peak at 1726 cm^− 1^ corresponds to the C = O stretching vibrations, typically from the carboxyl or carbonyl groups, and is known to enhance water solubility and provide sites for further functionalization. These groups also contribute to the fluorescence properties and play a role in the electron transfer interactions [75]. The peak at 1250 cm^− 1^ is associated with the C–O–C stretching vibrations, from epoxy or ether groups, and contributes to the stability, dispersion, and electronic interactions of CQDs [76]. Studies on citric acid-based CQDs have already been reported to have comparable FT-IR peaks. For instance, Dong et al., [75] have previously reported the O–H, C–H, and C = O groups for the CQDs synthesized from citric acid, with a peak around 3400 cm^− 1^ for O–H stretching, a peak near 2930 cm^− 1^ for C–H stretching, and a prominent 1720 cm^− 1^ peak for the C = O stretching. The FTIR analysis of LPE and LPCQDs demonstrated that most of the active functional groups present in the LPE were retained after carbonization conferring the bioactive properties to the synthesized LPCQDs (Table S3). Reports have demonstrated that the low temperature hydrothermal synthesis helps in preserving some of the functional groups from the precursor after carbonization [77], and these surface active functional groups play an essential part in enhancing the antibiofilm and antimicrobial properties of the carbon dots [78].

The TEM analysis of LPCQDs synthesized in the study has shown it to have a size range from 2 to 4 nm. CQDs in the observed size range could exhibit significant quantum confinement effects, responsible for the size-dependent optical and electronic properties. Quantum confinement often results in blue-shifted emission, where the smaller CQDs (closer to 2 nm) exhibit shorter wavelengths due to the increased band gap when compared to the same with larger CQDs (closer to 4 nm). The small size observed for the LPCQDs implies a large surface area relative to volume, favouring a greater proportion of the atoms to be present on the surface. This could further enhance the functionalization potential of LPCQDs, allowing for more surface functional groups to interact with the surrounding molecules or solvents, as required for the applications in sensing, bioimaging, and catalysis [76]. Previous studies have reported the CQDs synthesized by other physicochemical methods to have a size distribution of 2 to 10 nm. The CQDs with a 2–4 nm size range can have strong quantum confinement, resulting in blue or green emissions. In a previous study, Li et al. [79] have synthesized CQDs with an average size of 3 nm having blue-shifted emission, and the observation was in alignment with the results observed for the LPCQDs of the current study.

DLS spectrum has been used to measure the hydrodynamic diameter, which is the effective diameter of the particles when they interact with the solvent [80]. From the results of the study, LPCQDs were found to have a size of 2 nm, which suggests their well-dispersed nature in water, due to a uniform size distribution. The size consistency from this implies minimal aggregation, as the aggregated CQDs will have larger and broader size distributions. The stable dispersion of CQDs in distilled water without any significant aggregates could be due to the surface functional groups. These could execute the colloidal stability in water through the electrostatic and steric repulsion. Stability in aqueous media also indicates the suitability of the LPCQDs as desired for the biological and environmental applications, where water solubility and dispersibility are essential. In addition, DLS and TEM results of the study were found to align closely at around 2 nm, which is suggestive of a monodispersed and minimal aggregation nature of the LPCQDs as described before [81]. Previous reports on green-synthesised carbon dots have shown closely matching sizeranges as those reported from the TEM and DLS results of the current study, where the carbon dots are monodispersed with minimal aggregation [82] or in the case of carbon dots synthesized from lemon leaves [83] or from sugarcane juice [84]. Another review on the use of TEM and DLS clearly stated that proper synthesis procedures and sample preparation steps can help to reduce the mismatch in the size distribution data of the TEM and DLS of nanoparticles [85].

Zeta potential analysis has further revealed that the synthesized LPCQDs have an anionic nature with a mean zeta potential value of -0.5 mV. This could be due to the balance of surface functional groups such as carboxyl (-COOH) or hydroxyl (-OH) groups, which can dissociate in the aqueous environment, resulting in the anionic charge. As observed, the negative zeta potential of LPCQDs is relatively close to zero, which could be due to limited electrostatic repulsion [86]. Previous studies have attributed low negative values for green synthesized carbon dots due to the presence of hydrophilic functional groups on the surface of the carbon dots [87].

The carbon content analysis of LPCQDs is important to get information on the efficiency of conversion of lemon peel into carbon-rich quantum dots. From the CHNS results, a carbon content of 37% could be observed for the LPCQDs, which indicated its major mass to be contributed by the carbon, which is typical for the CQDs. This relatively high percentage supports the formation of a stable carbon core, which is essential for the effective PL properties. Earlier studies on the CQDs derived from various organic materials have already reported the carbon content to range from 30 to 80%, depending on the source and synthesis method. For instance, Sousa et al. [88] have previously reported a carbon content of about 51% for the CQDs derived from olive residues. This could also reflect the differences in the carbon conversion efficiency of materials used for the synthesis of CQDs.

The synthesized LPCQDs were also found to have excellent activity against clinically relevant pathogens like *E. coli*,* S. aureus*,* K. pneumoniae*,* P. mirabilis*, and *S. typhi*, with moderate antifungal activity against *C. albicans* and *A. flavus.* The MIC of LPCQDs was also observed to be 0.94 mg mL^−1^ against *E. coli*,* S. aureus*, and *S. typhi* and 1.88 mg mL^−1^ for *P. mirabilis* and *K. pneumoniae.* These results were comparable to the previous reports on MIC values of LPCQDs against bacterial pathogens [89]. The MFC of LPCQDs against *C. albicans* and *A. flavus* was observed to be 3.75 mg mL^−1^. However, the MBC of LPCQDs against all the selected bacterial strains was observed to be 3.75 mg mL^−1^. These results are indicative of the bioactivity of the CQDs, which could primarily be due to the presence of surface-active hydrophilic functional groups. Moreover, previous studies have also demonstrated the disordered arrangement of CQDs in the dispersion, favoring its damaging impact on the smooth outer membranes of microbial cells with the resulting antibacterial activity [90]. The presence of anionic groups, as indicated in the FTIR and zeta potential results, could also be supportive of the observed antimicrobial activity of LPCQDs as per previous reports [91]. The CQDs may also exert their antimicrobial properties through mechanisms such as ROS generation potential due to the presence of defects in the core structure, as evidenced by the Raman spectroscopic studies, resulting in injury to cellular components like proteins, lipid peroxidation, and nucleic acids which could ultimately cause microbial death [71]. Additionally, studies have shown that when microbial cells are exposed to CQDs for more than 24 h, the CQDs adhere to the cell wall, causing structural disruptions that would increase the permeability and ultimately compromise the cell integrity [92]. The FESEM images confirm cell wall and cell membrane disruption, and prior literature provides insights into the mechanism. Moreover, a recent investigation into the biophysical interaction of slightly anionic or neutral carbon dots with lipid membranes has demonstrated that CQDs cause unbalanced disruptions in the lipid bilayer, increasing the bilayer permeability, resulting in apoptosis [93].Earlier studies on CQDs from various sources have already reported comparable microbicidal efficacy, with variations in MIC and MBC as per the synthesis methods and functional modifications involved [94]. For instance, the CQDs functionalized with nitrogen or doped with metals like silver have been reported to have enhanced antibacterial activity, with the MIC values as low as 0.5 mg mL^−1^ [95]. Here, the lemon peel-derived CQDs were observed to have comparable activity without any additional doping or modification, which is suggestive of their potential to be used for diverse applications.

In the current study, the cytotoxicity of the LPCQDs was also evaluated against the L929 cell line by the MTT assay, which relies on the cellular reduction of MTT to a purple coloured compound-formazan by the mitochondrial dehydrogenases of the viable cells [96]. The mouse fibroblast L929 cell line, originating from connective tissues of C3H/An mouse, is a standard cell line for assessing cytotoxicity of carbon dots used in wound healing formulations [97]. The use of L929 has many advantages, such as rapid growth and easy growth under in-vitro conditions. It mimics the functions of fibroblast cells, which are the primary cells involved in wound healing and repairing tissues [98]. Moreover, it produces a significant amount of extracellular matrix proteins, which make the cell growth more rapid and are ideal for cell culture protocols. Here, the cell viability was observed to be 92% even with the use of 10 µg mL^−1^ of LPCQDs. The results obtained were concurrent with the previous reports on the minimal cytotoxicity of CQDs [99]; [100].

The LPCQDs used in the study were also found to have antibiofilm activity against *K. pneumoniae*, *S. aureus*, and *S. typhi.* This could be attributed to the higher penetrability of the CQDs into the biofilms, which thereby makes it a suitable candidate for eradicating the biofilms as described before [101]. AFM analysis could further provide deeper insight into the antibiofilm activity of LPCQDs against *K. pneumoniae*. In the positive control used here, a densely packed and rough biofilm structure with a height reaching up to 535.8 nm could be observed, due to the intact bacterial biofilm. In contrast, the bacterial cells treated with the LPCQDs were found to have a disrupted appearance with a significant reduction in height to 343.3 nm, and that too without the typical morphological features of biofilm. Reports have previously demonstrated the minuscule size of CQDs to be favourable for their penetration into the biofilm, easily disrupting the extracellular polymeric material [102]. The results hence showed the potential of LPCQDs to disrupt the biofilm stability, resulting in less organized and fragmented structures. The reduced biofilm thickness and disrupted cell aggregation observed with the LPCQD'-treated cells suggest that the quantum dots can interfere with the biofilm formation of *K. pneumoniae*, potentially by damaging the bacterial cell membranes or generating oxidative stress that interferes with the biofilm stability. Several reports have previously demonstrated CQDs to have promising biocompatibility along with high ROS generation, which makes them effective antibiofilm agents for medical devices and implants [103]. Future studies employing CLSM and EPS quantification can further elucidate the LPCQDs' antibiofilm mechanisms.

In the present study, LPCQDs have shown 96.01 ± 1.64% inhibition of BSA protein denaturation and 79.29 ± 0.53% for egg albumin and when compared to 96.21 ± 0.31% inhibition by diclofenac standard, a commonly prescribed non-steroidal drug for treating inflammation. Carbon dots have been previously known to inhibit protein denaturation due to the presence of surface-active hydrophilic groups, which can form hydrogen bonds and hydrophobic interactions with the protein skeleton, thereby covering up hydrophobic residues and blocking inflammatory cascades [40]. The structure of the BSA protein provides abundant sites for interaction with carbon dots, which leads to better penetration and stabilisation of the BSA proteins, further accounting for the higher performance of LPCQDs with the BSA. The lower values of inhibition for egg albumin could indicate substrate specificity, as egg albumin has disulfide bonds, which make the protein structure rigid and less penetrable to the carbon dots [104]. The CQDs are known for their anti-inflammatory properties due to their ability to inhibit protein denaturation. Additionally, the CQDs also have the potential to stabilize the protein structures by preventing the denaturation that could trigger inflammatory responses. In previous reports, CQDs synthesized from various natural sources, such as ginger and turmeric, were demonstrated to have comparable anti-inflammatory activities. The 90% inhibition of protein denaturation at 0.5 mg mL^− 1^, shown by the ginger derived CQDs, is supportive of the observed current results with the LPCQDs [105]. As the LPE itself has already been reported to have anti-inflammatory properties, the observed results indicate that LPCQDs were promising enough to retain the activity [106]. The findings in this study, hence, underscore the efficiency of LPCQDs, which are comparable to the previously studied CQDs, possibly due to the unique properties imparted by the lemon peel precursors.

The antidiabetic potential of LPCQDs is assessed through inhibition of α-amylase enzyme, an enzyme responsible for increased post prandial sugar levels in the blood. In the present study, the LPCQDs have shown 65.8 ± 5.85% α-amylase inhibition, which was significantly greater than the inhibition percentage (64.30 ± 0.77%) of the standard quercetin. Previous studies have shown carbon dots to possess antidiabetic potential, and it was elucidated that the phenolic compounds present in lemon peel-derived carbon dots have several functional groups that could bind to the active site of the amylase enzyme, thereby inhibiting enzyme activity through competitive inhibition [57]. Another study has attributed the anti-diabetic activity of nanodots to the abundance of structural defects in the core, which lead to electrostatic interactions that cause conformational changes in the enzyme structure, thereby inhibiting substrate binding [107]. The antidiabetic potential of LPCQDs could primarily be attributed to their ability to inhibit alpha-amylase, which is responsible for breaking down starch into glucose. In earlier reports, the inhibitory action has been attributed to the presence of hydroxyl groups exposed on the surface of CQDs and the ROS generation by CQDs, which could interfere with the binding sites of the amylase enzyme, and thereby its substrate binding [108]. Previous research has also reported that the CQDs synthesized using other sources also have promising alpha-amylase inhibitory activity. For instance, *Vitis vinifera* seeds derived CQDs have already been demonstrated to cause 80% inhibition of alpha amylase even at 50 µg mL^− 1^ [108]. In comparison, the LPCQDs appear to have a comparable inhibitory effect, which could be possible due to the lemon peel-derived structures present in them. This inhibition mechanism also provides a promising way to manage diabetes, as it targets glucose production at the digestive level rather than influencing insulin. Hence, the synthesized LPCQDs in this study can have the potential to be explored in developing new diabetes management therapies, either as dietary supplements or as active agents in functional foods.

CQDs have also been reported to have applications in metal detection. The most hazardous metal in the natural ecosystems is mercury, which is an unwanted by-product of the paper, refinery, rubber processing, and fertilizer industries. The US Environmental Protection Agency has designated mercury as one of its priority pollutants. For the release into inland and potable water, the concentration of Hg^2+^ ions must be below the tolerance level of 10 and 1 ppb, respectively. Chest discomfort, dyspnoea, renal impairment, and pulmonary dysfunction are the common toxicity symptoms associated with the ingestion of mercury [109]. With an ultralow detection limit, the LPCQDs synthesized in the study were found to be useful for application as a fluorescent probe for the detection of heavy metal ions, such as Hg^2+^ and Pb^2+^, in potable water. In fact, it is an in-situ method for identifying metal ions present in the aqueous medium through fluorescence quenching of LPCQDs. This method is user friendly and does not need the sophistication of common techniques like chromatography/mass spectrometry and enzyme-linked immunosorbent assays. The detection here focuses on creating a unique LPCQDs-based method to identify the metal ions from the aqueous solutions. LPCQDs here can be used as environment friendly sensors for the detection of heavy metal or metalloid pollutants in water. The renewability and efficient use of waste lemon peels make this an attractive green chemistry approach for the production of CQDs.

## Conclusion

The current study has deployed a facile method for the green synthesis of multifunctional CQDs from LPE. The synthesized LPCQDs were found to have diverse applications in pathogen inhibition, biofilm suppression, management of diabetes, and monitoring of heavy metal ions in the environment. The fluorescence quenching ability of the CQDs enabled them to detect micromolar levels of heavy metal ions, highlighting their potential in nano sensor research and environmental surveillance. Since the synthesised LPCQDs have shown superior antimicrobial and antibiofilm activities along with anti-inflammatory and antidiabetic properties, they can have the potential as an effective multifunctional therapeutic agent.

## Supplementary Information

Below is the link to the electronic supplementary material.

Supplementary Material 1

## Data Availability

No datasets were generated or analysed during the current study.
