# Supplementary material for: Ecofriendly hydrothermal synthesis of lemon peel derived multifunctional carbon quantum dots for biomedical and environmental sensing applications
Source: Discov Nano. 2026 Apr 13;21(1):119. doi: 10.1186/s11671-026-04524-7 (PMC13076827; doi:10.1186/s11671-026-04524-7)
Supplement: Supplementary file 1 — Supplementary Material 1 [file 11671_2026_4524_MOESM1_ESM.docx]

**Supplementary Data**

**Eco-friendly Hydrothermal Synthesis of Lemon Peel Derived Multifunctional Carbon Quantum Dots for Biomedical and Environmental Sensing Applications**


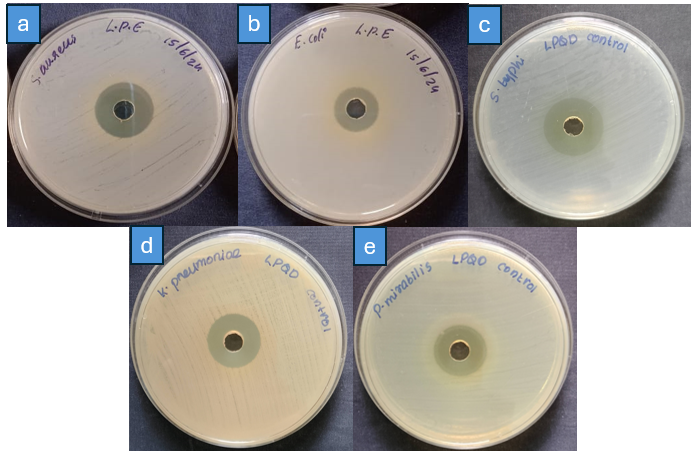


**Figure S1:** Antibacterial activity of LPE against (a) *S.aureus*, (b) *E. coli*, (c) *S.typhi,* (d) *K.pneumoniae*, and (e) *P.mirabilis.*


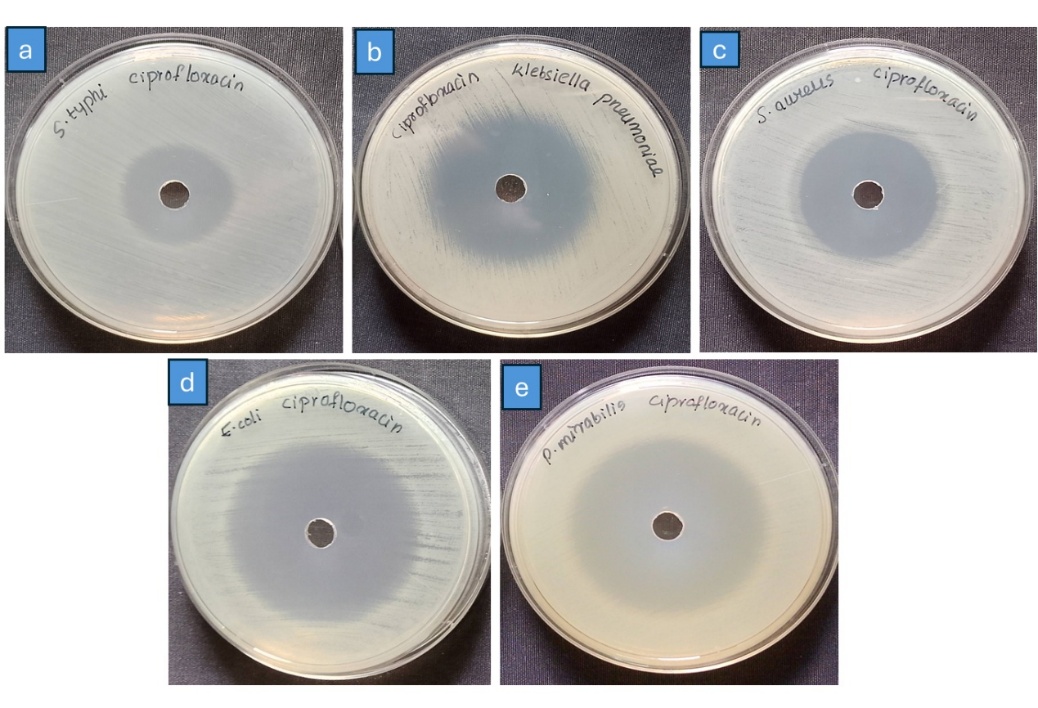


**Figure S2**: Antibacterial activity of Ciprofloxacin (positive control) against (a) *S.typhi*, (b) *K.pneumoniae*, (c) *S.aureus,* (d) *E. coli*, and (e) *P.mirabilis.*

**Table S1:** Comparison of MIC and MBC values of LPCQDs against *E. coli, S. aureus, K. pneumoniae, S. typhi,* and *P. mirabilis.*

| Conc. of LPCQD | Bacterial isolates | MIC (mg mL^-1^) | MBC (mg mL^-1^) |
| --- | --- | --- | --- |
| 15 mg mL^-1^ | *S. aureus* | 0.94 | 3.75 |
|  | *E. coli* | 0.94 | 3.75 |
|  | *K. pneumoniae* | 1.88 | 3.75 |
|  | *S. typhi* | 0.94 | 3.75 |
|  | *P. mirabilis* | 1.88 | 3.75 |


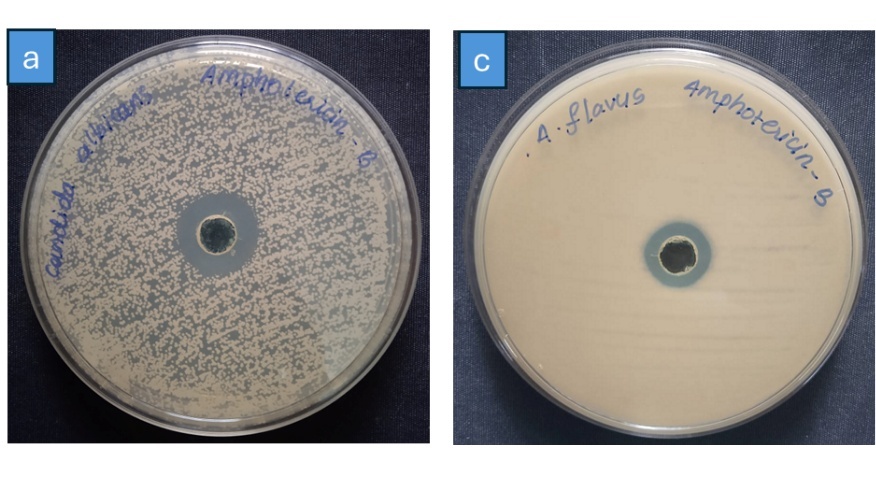


**Figure S3**: Antifungal activity of amphotericin B on (a) *Candida albicans* and (b) *Aspergillus flavus* using the disc diffusion method (n=3).


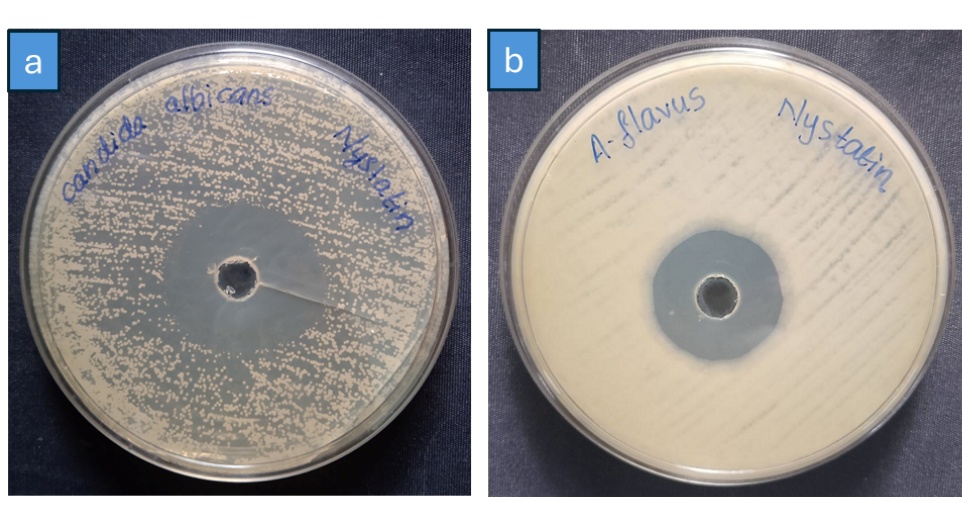


**Figure S4**: Antifungal activity of nystatin on (a) *Candida albicans* and (b) *Aspergillus flavus* using the disc diffusion method (n=3).


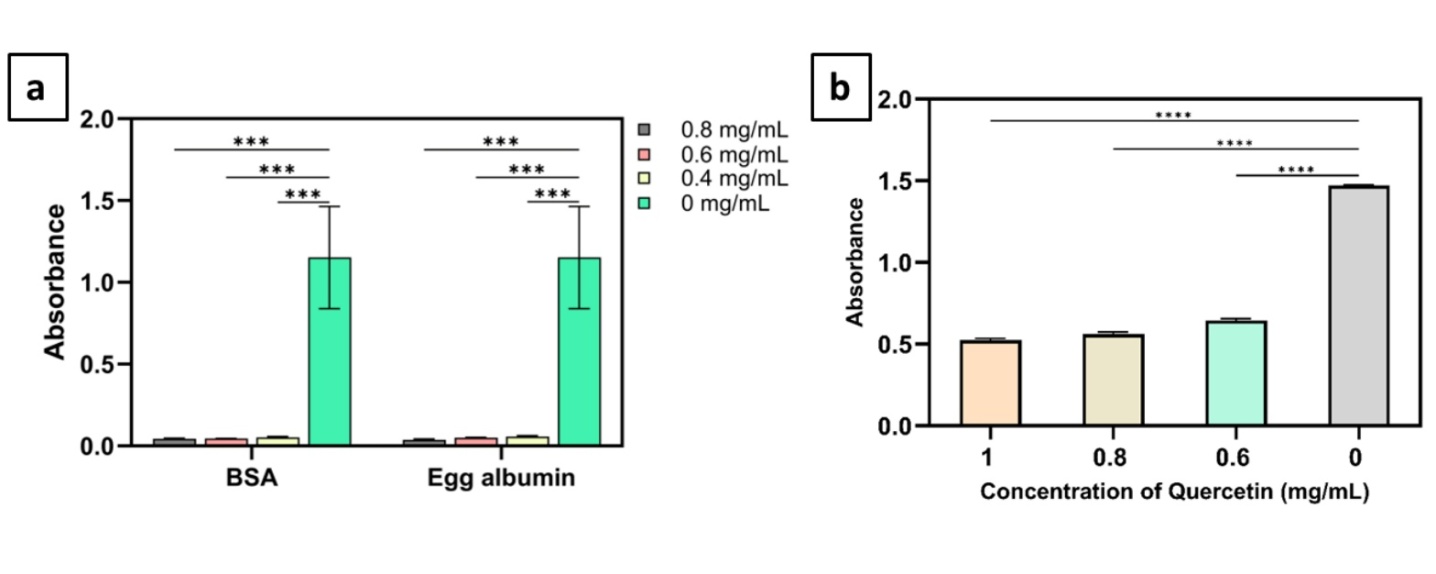


**Fig. S5** Effect of Standard drugs: (a) anti-inflammatory activity of diclofenac, and(b) antidiabetic activity of quercetin, p<0.05

**Table S2**: Comparative analysis of the LPCQDs with carbon dots previously reported in literature:

| **S.**  **No** | **Pretreatment**  **(Source)** | **Synthesis**  **method** | **Size (nm)** | **QY (%)** | **Cytotoxicity** | **Bioactivity** | **Sensing/Limit of detection** | **Reference** |
| --- | --- | --- | --- | --- | --- | --- | --- | --- |
| 1 | Sun drying and oven drying at 100 °C, treated with 0.1M Sulfuric acid, again dried at 100 °C, treated with Sodium hypochlorite for 4 h  (lemon peel wastes) | Hydrothermal 200°C/12 h | 1-3 | 14 | NR | NR | Cr^6+^/  73nM | [57] |
| 2 | Shade-drying, acid hydrolysis.  (fresh  lemon peels) | Hydrothermal 200°C/6h | 9.5 | 11 | NR | NR | Carmine | Su et al., 2018 [56] |
| 3 | 90°C drying, carbonized at 200 °C for 2 h in a muffle furnace  (Lemon peel powder) | Microwave pyrolysis 300W/5 min | 4.46 | 49.5 | NR | NR | Tetracycline | Kundu et al.,  (2023) [57] |
| 4 | 100°C drying, Aqueous extract  (Lemon bagasse) | Microwave pyrolysis (720W/5 min) | 3.1 | 10 | 85% (HFF1 cells) | NR | Antioxidant property | da Silva etal., 2023 [58] |
| 5 | Hot distilled water extract/domestic  Lemon peel waste | Microwave pyrolysis (900W/17 min) | NR | 0.81 | NR | NR | None | Medhi et al., 2025  [15] |
| 6 | Sun-drying, treated with Methanol,  Sulphuric acid, NaOCl, distilled water rinse  (Lemon peel zest) | Hydrothermal 200°C/12h | 2.18 | 3.1 | NR | NR | Antioxidant potential | Aouadi et. Al., 2025  [59] |
| **7** | ***Current study:***  **Shade dried at 45°C, Sonicated Ethanolic extract .**  **(Lemon peel waste from the market)** | **Hydrothermal, 180°C/8h** | **2.75** | **7.4** | **92% at (L929 cells)** | **Anti-inflammatory: 96%**, **Antidiabetic:65.8%, Antimicrobial against pathogens,**  **Antibiofilm 81.72%,** | **Hg²⁺/Pb²⁺ (700 µM)** | **Present Study** |

*NR-Not reported

**Table S3:** Peaks retained in LPCQDs from LPE

| **S no.** | **Peak wavenumber (cm^-1^)** | **Peaks in LPE** | **Peaks in LPCQDs** | **Peak assignments** | **References** |
| --- | --- | --- | --- | --- | --- |
| 1. | 3400 | 3375.43 | 3408.22 | O-H stretching; polyphenols | [7] |
| 2. | 2855 | 2895.15 | 2854.65 | Symmetric C–H stretching, aliphatic hydrocarbons | [8] |
| 3. | 1726 | 1726.29 | 1726.29 | C=O (carboxyl / ester) | [9] |
| 4. | 1600 | 1606.70 | 1614.42 | C=C / amide I | [10] |
| 5. | 1240 | 1230.58 | 1205.51 | C-O bond stretching, esters | [11] |
| 6. | 1100 | 1095.57 | 1087.85 | C-O-C stretching, sugars | [12] |
| 7. | 768 | 759.95 | 767 | Ring deformation | [13] |

**References :**

[1] A. Tyagi, K. Malika Tripathi, N. Singh, S. Choudhary, R. Kumar Gupta, Green synthesis of carbon quantum dots from lemon peel waste: applications in sensing and photocatalysis, RSC Advances 6 (2016) 72423–72432. https://doi.org/10.1039/C6RA10488F.

[2] A. Su, D. Wang, X. Shu, Q. Zhong, Y. Chen, J. Liu, Y. Wang, Synthesis of Fluorescent Carbon Quantum Dots from Dried Lemon Peel for Determination of Carmine in Drinks, Chem. Res. Chin. Univ. 34 (2018) 164–168. https://doi.org/10.1007/s40242-018-7286-z.

[3] A. Kundu, S. Basu, B. Maity, Upcycling Waste: Citrus limon Peel-Derived Carbon Quantum Dots for Sensitive Detection of Tetracycline in the Nanomolar Range, ACS Omega 8 (2023) 36449–36459. https://doi.org/10.1021/acsomega.3c05424.

[4] L.E. da Silva, O.L. de L. Calado, S.F. de Oliveira Silva, K.R.M. da Silva, J. Henrique Almeida, M. de Oliveira Silva, R. da S. Viana, J.N. de Souza Ferro, J. de Almeida Xavier, Cintya.D.A.E.S. Barbosa, Lemon-derived carbon dots as antioxidant and light emitter in fluorescent films applied to nanothermometry, Journal of Colloid and Interface Science 651 (2023) 678–685. https://doi.org/10.1016/j.jcis.2023.07.124.

[5] M. Medhi, M. Yumnam, P. Mudoi, P. Mishra, Green florescent carbon dots synthesized from various household green wastes for detection of parathion methyl pesticide, Journal of Luminescence 277 (2025) 120926. https://doi.org/10.1016/j.jlumin.2024.120926.

[6] A. Aouadi, D.H. Saoud, A. Bouafia, H.A. Mohammed, H.G. Gamal, A. Achouri, S.E. Laouini, M.M.S. Abdullah, B.M. Al-maswari, H.A. Al-Lohedan, Unveiling the antioxidant power: synthesis and characterization of lemon and orange peel-derived carbon quantum dots with exceptional free radical scavenging activity, Biomass Conv. Bioref. 15 (2025) 9691–9704. https://doi.org/10.1007/s13399-024-05765-1.

[7] N. Kitadai, T. Sawai, R. Tonoue, S. Nakashima, M. Katsura, K. Fukushi, Effects of Ions on the OH Stretching Band of Water as Revealed by ATR-IR Spectroscopy, J Solution Chem 43 (2014) 1055–1077. https://doi.org/10.1007/s10953-014-0193-0.

[8] K. Forfang, B. Zimmermann, G. Kosa, A. Kohler, V. Shapaval, FTIR Spectroscopy for Evaluation and Monitoring of Lipid Extraction Efficiency for Oleaginous Fungi, PLoS One 12 (2017) e0170611. https://doi.org/10.1371/journal.pone.0170611.

[9] J.M. Cervantes-Uc, J.V. Cauich-Rodríguez, H. Vázquez-Torres, A. Licea-Claveríe, TGA/FTIR study on thermal degradation of polymethacrylates containing carboxylic groups, Polymer Degradation and Stability 91 (2006) 3312–3321. https://doi.org/10.1016/j.polymdegradstab.2006.06.005.

[10] F.A. Miller, Amides, Carboxylate Ion, and CO Single Bonds, in: Course Notes on the Interpretation of Infrared and Raman Spectra, John Wiley & Sons, Ltd, 2004: pp. 205–215. https://doi.org/10.1002/0471690082.ch8.

[11] Y. Dong, H. Pang, S. Ren, C. Chen, Y. Chi, T. Yu, Etching single-wall carbon nanotubes into green and yellow single-layer graphene quantum dots, Carbon 64 (2013) 245–251. https://doi.org/10.1016/j.carbon.2013.07.059.

[12] M. Margoshes, V.A. Fassel, The infrared spectra of aromatic compounds: I. The out-of-plane C-H bending vibrations in the region 625–900 cm−1, Spectrochimica Acta 7 (1955) 14–24. https://doi.org/10.1016/0371-1951(55)80003-3.

[13] X. Li, Y. Ju, Q. Hou, Z. Li, J. Fan, FTIR and Raman Spectral Research on Metamorphism and Deformation of Coal, Journal of Geological Research 2012 (2012) 590857. https://doi.org/10.1155/2012/590857.
